# A sulfur-containing volatile emitted by potato-associated bacteria confers protection against late blight through direct anti-oomycete activity

**DOI:** 10.1038/s41598-019-55218-3

**Published:** 2019-12-30

**Authors:** Delphine Chinchilla, Sébastien Bruisson, Silvan Meyer, Daniela Zühlke, Claudia Hirschfeld, Charlotte Joller, Floriane L’Haridon, Laurent Mène-Saffrané, Katharina Riedel, Laure Weisskopf

**Affiliations:** 1 Department of Biology, University of Fribourg, Chemin du Musée 10, CH-1700 Fribourg, Switzerland; 2 Department of Microbial Physiology and Molecular Biology, University of Greifswald, Felix-Hausdorff-Strasse 8, D-17489 Greifswald, Germany; 3 Department of Microbial Proteomics, University of Greifswald, Felix-Hausdorff-Strasse 8, D-17489 Greifswald, Germany

**Keywords:** Plant immunity, Plant stress responses, Bacteria, Pathogens, Fungal physiology, Antifungal agents, Proteomics

## Abstract

Plant diseases are a major cause for yield losses and new strategies to control them without harming the environment are urgently needed. Plant-associated bacteria contribute to their host’s health in diverse ways, among which the emission of disease-inhibiting volatile organic compounds (VOCs). We have previously reported that VOCs emitted by potato-associated bacteria caused strong *in vitro* growth inhibition of the late blight causing agent *Phytophthora infestans*. This work focuses on sulfur-containing VOCs (sVOCs) and demonstrates the high *in planta* protective potential of S-methyl methane thiosulfonate (MMTS), which fully prevented late blight disease in potato leaves and plantlets without phytotoxic effects, in contrast to other sVOCs. Short exposure times were sufficient to protect plants against infection. We further showed that MMTS’s protective activity was not mediated by the plant immune system but lied in its anti-oomycete activity. Using quantitative proteomics, we determined that different sVOCs caused specific proteome changes in *P. infestans*, indicating perturbations in sulfur metabolism, protein translation and redox balance. This work brings new perspectives for plant protection against the devastating Irish Famine pathogen, while opening new research avenues on the role of sVOCs in the interaction between plants and their microbiome.

## Introduction

In nature, plants are exposed to many different types of stress, one of which is the attack by disease-causing agents that encompass viruses, bacteria, fungi and oomycetes. *Phytophthora infestans*, an oomycete causing late blight in potato and tomato, is one of the most devastating pathogens worldwide due to its fast asexual life cycle and to the occurrence of sexual reproduction leading to genetically diverse populations^1^. The fast disease spread is facilitated by the massive production of two types of asexual spores: sporangia that can be dispersed by wind and rainfall, and zoospores, which are motile, bi-flagellated spores able to swim towards stomata or daughter tubers^1^. To control late blight, most growers rely on repeated applications of synthetic or copper-based fungicides, but the fast emergence of fungicide-resistant *P. infestans* strains^2^ and the side-effects of these compounds on environment and human health urge the search for alternative disease-control strategies. Resistance breeding is one of them, but its success is threatened by the ability of *P. infestans* to quickly overcome resistance genes^1,2^. Beyond resistance encoded in the plant’s own genetic makeup, recent reports indicate that the plant microbiome, i.e. the microbes living in close association with the plant, might contribute to the defence of their host against pathogens^3,4^. In an attempt to exploit this protective potential, we isolated bacterial strains from the phyllosphere and rhizosphere of potato and characterized their protective activity against late blight^5–8^. Plant-associated bacteria are known to promote plant growth and health by a wide range of processes, including niche competition, direct antibiosis, or stimulation of plant defences in a process called Induced Systemic Resistance (ISR)^9–11^. Recently, the ability of plant-associated bacteria to emit volatile organic compounds (VOCs) has emerged as an important determinant of their promoting effect on plant growth and health^12–15^. Some of these bacterial VOCs have been shown to act directly on plant pathogens^16^, while others have been reported to induce ISR^17,18^. In earlier work, we characterized the volatilomes (i.e. the blends of VOCs) emitted by our collection of potato-associated *Pseudomonas* with strong inhibitory activity against *P. infestans*. In addition to hydrogen cyanide, we identified sulfur-containing volatiles (sVOCs) as potent inhibitors of the oomycete’s *in vitro* growth^5,19^. In contrast to elemental sulfur, which has long been used in crop protection against fungi^20^, the discovery that volatile organic sulfur compounds also have strong crop protection potential is more recent. Dimethyl disulfide (DMDS), which is produced by many bacteria^21^ and by some plant species such as *Alliaceae*^22^ and *Brassicaceae*^23^, has received most attention and is being used in practice for soil fumigation against weeds, nematodes and pathogenic fungi^24,25^. However, in our *in vitro* characterization of the biological effect of bacterial sVOCs on different life stages of *P. infestans*, the protective activity of DMDS was largely surpassed by that of two others sVOC, dimethyl trisulfide (DMTS) and S-methyl methane thiosulfonate (MMTS)^19^. This latter compound, which is also produced by *Brassicaceae* such as cabbage, cauliflower or broccoli, and by *Liliaceae* such as garlic^26^, maintained high *in vitro* inhibition potential on all tested life stages of *P. infestans* even in very low concentrations^19^, which raised the questions of its suitability as new plant protection product and of its mode(s) of action on plant and pathogen. The aims of the present study were therefore i) to investigate the protective potential of MMTS and other selected sVOCs *in planta* using both potato leaf discs and plantlets, ii) to determine whether these sVOCs induced plant defences and/or acted directly on the pathogen, and iii) to define possible biological targets in *P. infestans*.

## Results and Discussion

### Sulfur-containing volatile organic compounds constrain late blight in potato leaf discs

**Figure 1:**
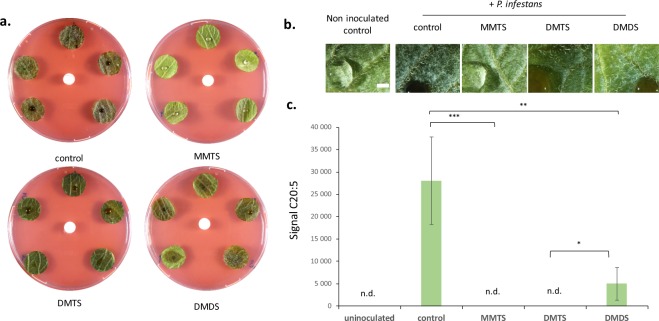
Sulfur-containing VOCs restrain late blight disease in potato leaf discs. (**a**) Leaf discs from Bintje adult plants (n = 5) were inoculated with *P. infestans* (Rec01) and simultaneously exposed to 1 mg MMTS, DMTS, or DMDS (or solvent used as control) loaded on a central silicone septum. Pictures are shown after 6 days of incubation and are representative of 3 independent assays. (**b**) Binocular pictures of co-treated leaf discs as described. Scale bar = 1 mm. (**c**) Quantification of oomycete infection by dosage of *P. infestans* fatty acids in leaf samples. Significant differences according to an ANOVA test are marked by asterisks: *p < 0.05; **p < 0.01 and ***p < 0.001. n.d. = not detected.

Following an initial screen for *P. infestans-*inhibiting VOCs that revealed the high *in vitro* activity of sulfur-containing volatiles (sVOCs)^19^, we explored the capacity of three sVOCs, DMDS, DMTS and MMTS (see Fig. S1 for the chemical structures of these sVOCs) to inhibit late blight *in planta* using leaf disc assays. Airborne exposure to 1 mg of DMTS or MMTS in the Petri dish atmosphere (80 mL) led to full protection against *P. infestans*, while DMDS was by far less active (Fig. 1a). Binocular observation confirmed that MMTS and DMTS totally prevented the development of *P. infestans* at the leaf surface (Fig. 1b). Nevertheless, we could not exclude at this stage that internal leaf tissues might be colonized by the pathogen. We therefore used a fatty acid methyl esters (FAMEs) analysis to quantify the oomycete in plant tissues. *P. infestans* produces specific fatty acids, such as the eicosapentaenoic acid (EPA; C20:5)^27,28^ that may serve as molecular markers to quantify the oomycete biomass in plant tissues, as previously demonstrated for *P. sojae* or *Plasmopara viticola*^29,30^. FAME analysis of inoculated leaf discs revealed several fatty acids that were specifically detected in heavily infested samples (Fig. S2a). A major peak confirmed by GC-MS analysis as C20:5 (Fig. S2b) was used to quantify the pathogen in the different treatments. Our results showed that MMTS and DMTS totally prevented the proliferation of *P. infestans* in potato leaf discs, while DMDS only partially prevented it (Fig. 1c).

**Figure 2:**
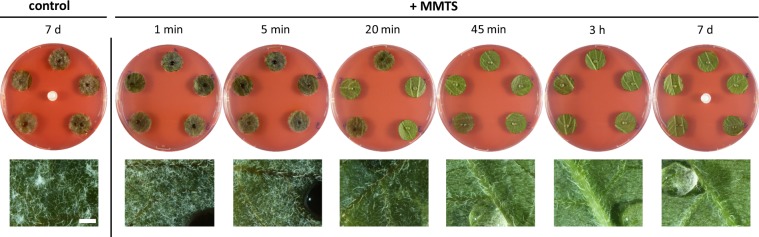
The inhibitory effect of MMTS on late blight over time. Leaf discs from Bintje adult plants (n = 5) were inoculated with *P. infestans* and simultaneously exposed to 1 mg MMTS (or solvent used as control). At different time points (1 min to 3 h), the septum carrying the volatile was removed to stop the volatile treatment. Pictures are shown after six days of incubation and are representative of three technical replicates. Binocular pictures taken from the first disc of each box are shown below. Scale bar = 1 mm.

**Figure 3:**
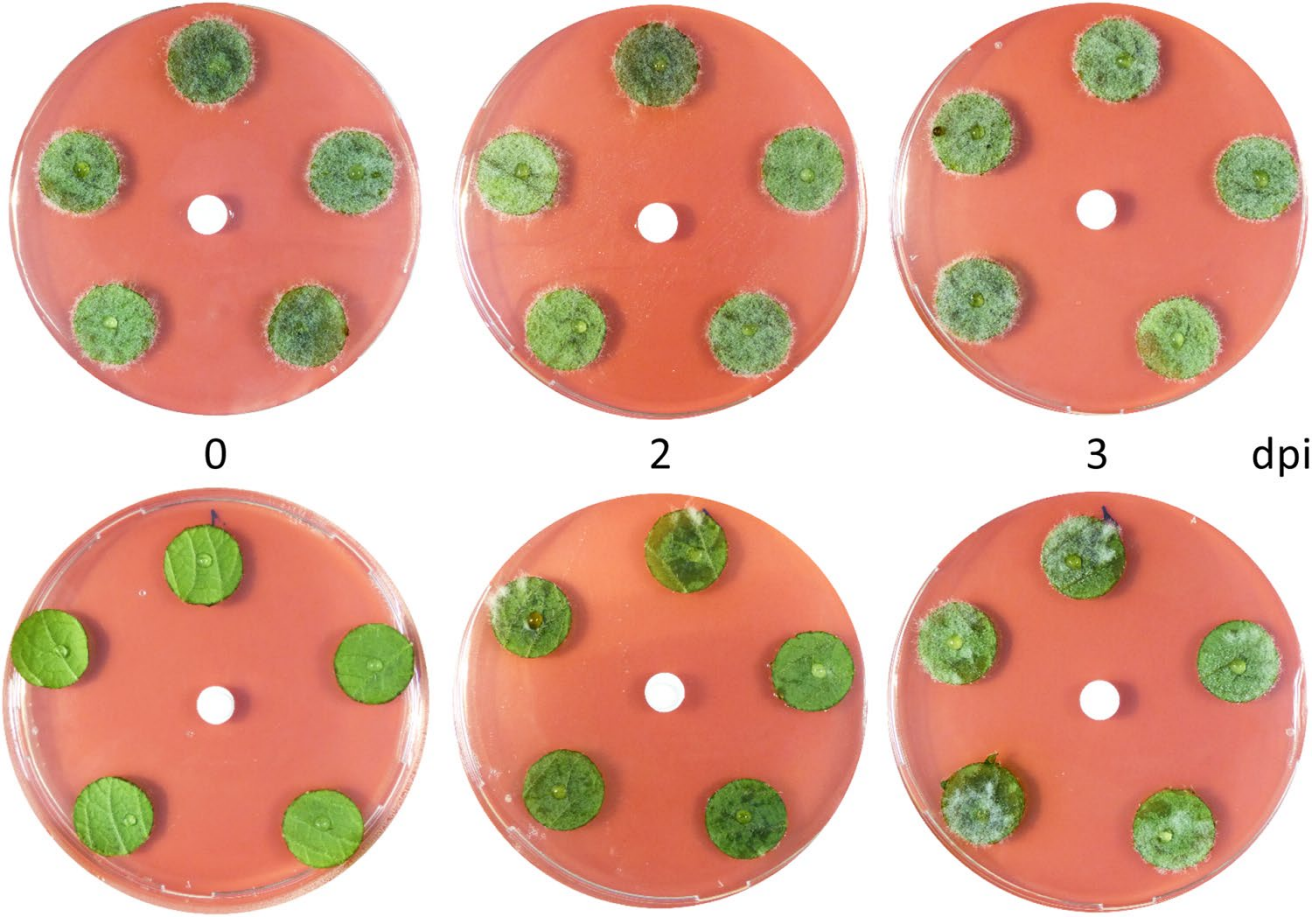
MMTS protects potato leaf discs against late blight when applied post infection. Leaf discs were infected with *P. infestans* at day 0 and further treated with solvent (upper panel) or 1 mg MMTS (lower panel) at 0, 2 and 3 days post infection (dpi). Pictures are shown after six days of incubation and are representative of three technical replicates. The biological experiment was repeated twice.

We also examined the phenotype of the sVOC-treated leaf discs without pathogen. Apart from natural colour variation possibly due to differing anthocyanin contents, the DMDS- and especially DMTS-treated leaf discs exhibited toxicity symptoms including dark colour and water soaking (Fig. S3). In contrast, MMTS induced no or very little visible damage (Fig. 1 and Fig. S3) and conferred an efficient protection against late blight even at lower dose, i.e. 100 μg per Petri dish (Fig. S4), which corresponds to 1.25 mg.L^−1^ of air. Moreover, a time-course experiment revealed that a 20 min treatment was already efficient to restrict late blight, which fully disappeared after 45 min of exposure to MMTS (Fig. 2). In practice, copper-based fungicides are commonly used to prevent late blight disease, but they only act preventively^31^. To assess whether MMTS was solely preventive too, we applied this sVOC once *P. infestans* infection had started. Interestingly, we observed strong disease reduction when MMTS was applied two days after *P. infestans*, indicating a potential for curative action (Fig. 3).

### MMTS inhibits late blight development in potato plantlets

**Figure 4:**
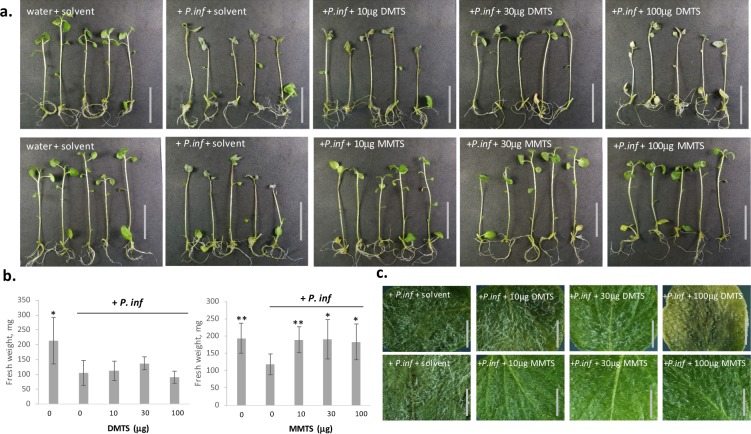
MMTS protects potato plantlets against late blight. **(a)** *In vitro* grown potato plantlets (cv Victoria) were inoculated with *P. infestans* and treated (from left to right) with 0, 10, 30 and 100 µg DMTS (upper panel) and MMTS (lower panel) respectively. Scale bar = 5 cm. (**b**) The fresh weight was measured for the respective plantlets. The bars show averages of ten individual plantlets with error bars indicating standard deviation. Significant differences between the infected controls and the samples according to an ANOVA test are marked by asterisks: *p < 0.01; and **p < 0.001. (**c**) Representative pictures taken at the binocular. Scale bars = 3 mm.

Next, we tested the protection efficiency of sVOCs in whole plants, using *in vitro* potato plantlets. Applying *P. infestans* zoospores on one leaf of potato plantlets led to successful infection, evidenced by wilting and by a white mat of hyphae and sporangiophores on the stem and on all leaves (Fig. 4). By contrast, plantlets treated with 10 to 100 μg MMTS in the tube atmosphere (40 mL) showed no late blight symptom, which also resulted in higher biomass than the non-treated, infected controls (Fig. 4b). Here too, MMTS had no phytotoxic effect and leaves showed a normal phenotype under binocular and microscopic examination. In contrast to MMTS, 30 μg DMDS and DMTS induced only limited disease protection (Fig. 4 and Fig. S5). At higher doses, DMTS was highly phytotoxic, inducing arrested growth and bleaching (Fig. S6), which confirmed earlier observations on leaf discs. The structurally related DMDS did not induce visible toxicity symptoms and both sulfides induced slight – but non-significant – plant growth promotion at 10 μg (Fig. S6b). Finally all sVOCs applied at 1 mg/tube induced strong phytotoxicity, indicating that a proper adjustment of MMTS dosage is required to balance plant protection vs. plant fitness (data not shown). Under these experimental conditions, the minimal active dose of MMTS was 1.75 mg.L^−1^ air. However, since the glass tubes represented a high humidity environment particularly conducive to late blight, we investigated whether lower doses would be sufficient in a lesser artificial setup. Indeed, when *in vitro* plantlets were transferred to pots and incubated in plastic boxes, 1 mg MMTS was sufficient to fully inhibit disease symptoms and did not induce any phytotoxicity (data not shown). This corresponds to a dose of 0.24 mg.L^−1^ air.

In view of the emergence of fungicide resistance in *P. infestans* strains, it is urgent to find new solutions to control this versatile pathogen^2^. Here, we show that the sVOC MMTS diffuses through the air and inhibits infection at doses that are not toxic to plants. Importantly, short exposure to MMTS can stop late blight development and this compound even shows protective potential when applied after inoculation of the pathogen. In order to better understand the mode of action of this efficient late blight inhibitor, we next investigated whether the protection originated form direct anti-oomycete activity or from an induction of plant defences.

### Protection against pathogens conferred by MMTS is independent from plant defences

**Figure 5:**
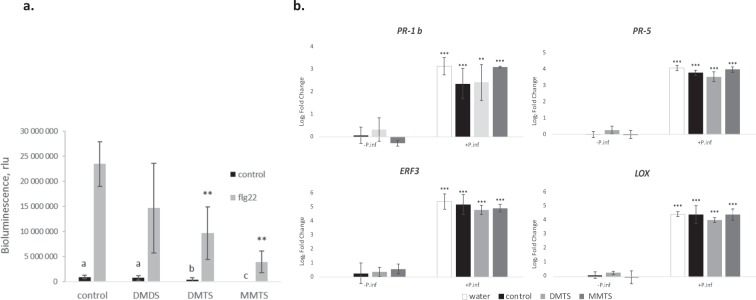
The effect of sVOCs on plant defence responses. (**a**) The effect of individual sVOCs (1 μg) on the flg22-induced oxidative burst in Arabidopsis, measured as the occurrence of bioluminescence from the oxidation of luminol by peroxidase. The bars show averages of six replicates with error bars indicating standard deviation. Significant differences toward control samples according to an ANOVA test are marked by asterisks or letters: **p < 0.001. These experiments were performed twice with similar results. (**b**) The accumulation of transcripts for defence genes as indicated in the graphs was analyzed by quantitative PCR in potato leaf discs in response to a 6 h treatment with 1 mg of MMTS or solvent control in the absence (−Pinf) or presence (+Pinf) of *P. infestans*. The bars show averages of 3 biological replicates with error bars indicating standard deviation. Significant differences toward control samples according to an ANOVA test are marked by asterisks: **p<0.01, ***p<0.001.

Many beneficial plant-associated microbes, including *Pseudomonas*, protect plants against pathogens by triggering ISR^11^. Microbe-associated molecular patterns (MAMPs) are recognized by plants and induce plant defences. Among other bacterial determinants, volatiles (e.g. 2,3- butanediol) were shown to induce the expression of defence genes in Arabidopsis^17,32^. Giving the strong protective effect of MMTS against late blight, we assessed whether this volatile elicited the plant defence responses. First, MMTS was applied two days before the pathogen to allow putative induction of defences. This preventive treatment did not lead to a lesser infection, suggesting that MMTS did not induce plant defences in this experimental setup (Fig. S7). Next, we investigated whether sVOCs induced the accumulation of reactive oxygen species (ROS) in plant tissues. MAMP perception by plant cells induces an “oxidative burst”, i.e. a rapid and transient accumulation of ROS^33^. We applied a luminol‐based chemiluminescent assay to detect ROS production and used the synthetic peptide flg22 (from flagellin) as positive control of MAMP response^34^. Preliminary assays with potato leaf discs showed a large inter-replicate variation and we therefore performed this analysis on Arabidopsis. Leaf discs treated with 1 μg MMTS, DMTS and DMDS exhibited no detectable increase in luminescent signal, such as the one observed with flg22 (Fig. 5a). We concluded that these sVOCs did not trigger the typical MAMP-induced oxidative burst. Interestingly, the samples treated with DMTS and MMTS showed significant luminescence reduction after flg22 treatment (Fig. 5a). This effect was dose-dependent and specific for these two sVOCs, as DMDS and another sulfur volatile, bis(methylthiomethyl) sulfide (BMTMS) had no significant effect on the flg22-induced oxidative burst (Fig. S8).

As sVOCs might easily oxidize^35^, we suspected that they compromised the chemical reaction of luminol oxidation used to detect ROS production. In assays where the various sVOCs were supplemented few minutes after elicitation with flg22, DMTS and MMTS (but not DMDS) quickly decreased the luminescent signal (Fig. S9). The question if sVOCs directly affected the oxidation reaction remains open, as we cannot exclude that these compounds might exhibit some toxicity to plant cells when applied directly into solution. Nevertheless, this finding is interesting as previous studies have proposed that sulfane sulfur (sulfur atoms that are bonded covalently in chains to other sulfur atoms) volatiles might carry antioxidant properties^36^. Our current data indicate that the sulfane sulfur-carrying DMTS and MMTS might interfere directly with the ROS produced by plant cells upon flagellin perception.

To further investigate the potential of these two protective sVOCs to activate plant immunity, transcript levels of defence-related genes were analysed in potato leaf discs exposed or not to sVOCs. Genes encoding Ethylene Response Factor 3 (ERF3), linoleate 9S-lipoxygenase 2-like (LOX), Pathogenesis-related protein 1b precursor (PR1-b), and Thaumatin-like protein (PR-5) were selected as defence markers as previously described in potato^37^. When applied without *P. infestans*, MMTS and DMTS did not induce significant changes in transcript levels compared with the controls. While gene expression increased significantly upon infection with *P. infestans*, it was similar in control and volatile-exposed leaf discs (Fig. 5b), showing that MMTS and DMTS did not affect the expression of defence-related genes in potato. Altogether, our data point to the conclusion that the protection conferred by MMTS against last blight is not mediated by the typical plant defence pathways, but rather by a direct anti-oomycete activity.

### Global changes in the Phytophthora proteome after sVOC- treatment

A quantitative proteomic approach was used to get insights into the biological pathways affected by MMTS and other sVOCs on *P. infestans*. In this experiment, we compared the proteome changes induced by 24 h exposure to 300 μg of each of five individual sVOCs detected in the volatile blends of potato-associated *Pseudomonas* and differing in their anti-oomycete activity^19^: MMTS, DMTS, DMDS, bis(methylthiomethyl) sulfide (BMTMS), and S-methyl butanethioate (SM). DMTS and MMTS led to strong inhibition of *P. infestans* mycelial growth, while the effect was less strong for BMTMS and only marginal for DMDS and SM (Fig. S10)^19^. Stringent quality filters were applied and only proteins identified in at least two of three biological replicates were considered. We detected 3348 *P. infestans* unique proteins, corresponding to 19% of the total proteome (Supplementary Table 1). Label-free quantification allowed to semi-quantitatively assess their expression and identify “regulated proteins”, i.e. those detected in lower or higher amounts in the treatment vs. control samples, with two-fold change used as threshold (Supplementary Table 1).

**Figure 6:**
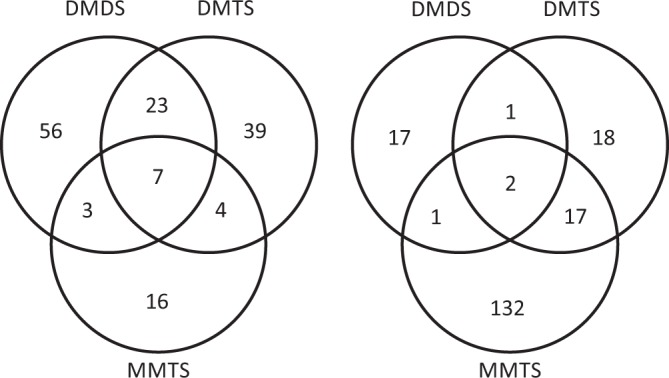
Overview of proteins commonly or specifically regulated by individual sVOCs. Venn diagrams depict the comparison of proteins regulated by DMDS, DMTS and/or MMTS in *P. infestans*. Left, UP/ON proteins; right, DOWN/OFF proteins.

Similar proportions of proteins were found regulated by DMDS and DMTS (around 3.3%) on the one hand, and by BMTMS, SM and MMTS (4.5 to 5.4%) on the other hand. A striking observation was that 80% of the MMTS-regulated proteins were downregulated or undetectable, which likely reflects the strong anti-oomycete activity of this volatile. This massive downregulation contrasts with the effect of DMDS, which mainly induced upregulation of proteins. We observed strong specificity in the proteome changes caused by exposure to the individual sVOCs, with only few proteins commonly regulated by DMDS, DMTS and MMTS (Fig. 6) or by the 5 sVOCs (data not shown). This specificity is consistent with their differential activities on hyphae and spores^19^. A larger proportion of shared upregulated proteins were found between DMTS and DMDS (n = 23) than between MMTS and DMDS (n = 3) or between MMTS and DMTS (n = 4) (Fig. 6). By contrast, the overlap of downregulated proteins was low between DMDS and DMTS, but higher between DMTS and MMTS, which again might relate to their stronger anti-oomycete activity.

The KOG (euKaryotic Orthologous Groups) database was used for functional classification of all identified proteins. Proteins were grouped into 26 categories according to their putative functional classes (Supplementary Table 1). Between 30% (BMTMS) and 42% (DMDS) of all identified regulated proteins had no functional assignment, which might be linked to the poor functional annotation of the *P. infestans* proteome. Most of the others had putative functions associated with i) intracellular trafficking, secretion, and vesicular transport (predominant class for all sVOCs except DMDS), i) post-translational modification, protein turnover, chaperones (predominant for all sVOCs except DMTS), iii) signal transduction (predominant for DMDS, DMTS, MMTS), and iv) transcription. Voronoi treemaps illustrating global changes in protein expression patterns upon exposure to each of the five sVOCs are shown as supplementary data. In general, no dramatic increase/decrease in proteins enabled to point to specific biological processes (max induction fold: 5; max reduction fold: 0.07), although differences were statistically significant (Supplementary Table 1). These results might indicate that sVOCs have multiple targets in *P. infestans*, as suggested for the sulfur sulfane structure shared by DMDS, DMTS and MMTS^35^. Nevertheless, some functional classes were more specifically affected by individual sVOCs, such as “amino-acid metabolism” for DMTS and “translation, ribosomal structure and biogenesis” for BMTMS. The biological relevance of the changes detected after BMTS, DMTS and MMTS exposure is discussed below.

### BMTS treatment affects the abundance of proteins involved in ribosome biogenesis

The most obvious specific effect of individual sVOCs on the *P. infestans* proteome occurred upon exposure to the moderately active BMTMS^19^ (Fig. S10). Manual inspection of BMTMS-regulated proteins and Gene Ontology enrichment analysis revealed a group of proteins related to ribosomes (GO:0003735 Structural constituent of ribosome; GO:00006412 Translation) (Table 1). The ribosome of *P. infestans* is composed of two subunits (40S and 60S)^38^. Five 60S proteins and two 40S proteins showed decreased abundance upon exposure to BMTMS, but not to other sVOCs. Each subunit comprises proteins associated to ribosomal RNAs (rRNA). Ribosomal RNA are modified by pseudouridylation, which is thought to regulate the stability and translational function of the ribonucleoprotein complex^39^. BMTMS affected the quantity of three important members of the H/ACA ribonucleoprotein complex, involved in the pseudouridylation of rRNA. The link between these and the regulated 40S/60S proteins remains unclear, but we could assume that if the ribonucleoprotein complex is less stable due to impaired rRNA pseudouridylation^39^, other components might be incorrectly stabilized and thus degraded by the cell. Altogether, our data suggest that BMTMS inhibits protein translation. This finding is of particular interest in view of the higher translation activity observed at particular, infection-relevant stages of the oomycete cycle: germinating cysts of *P. sojae* and *P. ramorum* exhibited a strong increase in proteins involved in ribosome structure, biogenesis and translation^40^ and similar observations were reported for the fish pathogen *Saprognelia parasitica*^41^. This increased translation activity may also represent the requirement to build the necessary machinery for host invasion, e.g. appressoria or effectors. In this respect, identifying a natural compound interfering with such processes might open promising research avenues. Although BMTMS had only modest effects on *P. infestans* development *in vitro*^19^ (Fig. S10), it might be interesting to re-analyse its activity on the ability of zoospores to encyst on plant tissue. If verified, such partial protective effect might be valuable enough in combination with other compounds of differing modes of action, e.g. acting on mycelial growth or sporulation.

**Table 1:** BMTMS-regulated proteins involved in ribosomal function.

Specificity	ID	Fold change	Molecular function	Biological process
	D0NR65 PITG_15407	0.074	60S ribosomal protein L34	Large subunit 60S^a^
	D0NY29 PITG_18052	0.114	60S ribosomal protein L3 and related proteins	Large subunit 60S^a^
	D0N4E3 PITG_06237	0.183	60S ribosomal protein L2/L8	Large subunit 60S^a^
	D0NG62 PITG_11099	0.21	60S ribosomal protein L18	Large subunit 60S^a^
	D0MU72 PITG_01833	0.223	60S ribosomal protein L13a	Large subunit 60S^a^
	D0NFC2 PITG_10450	OFF	putative ribosomal S6 kinase	Regulation of the small subunit 40S
	D0NLP0 PITG_13312	2.06	40S ribosomal protein S25	Small subunit 40S^a^
	D0P0D1 PITG_19608	2.05	Box H/ACA small nucleolar RNP component (NHP2)	Pseudouridylation of ribosomal RNA^b^
	D0N4G5 PITG_06263	OFF	H/ACA ribonucleoprotein complex subunit 4/ pseudo-uridine synthase (DKC1)	Pseudouridylation of ribosomal RNA^b^
*OFF with MMTS*	D0NS88 PITG_15652	OFF	H/ACA small nucleolar RNP component (GAR1)	Pseudouridylation of ribosomal RNA^b^

^a^KEGG pif03010: Ribosome.

^b^KEGG pif 03008: Ribosome biogenesis.

### Proteins of the sulfur metabolism are differentially regulated upon DMTS and MMTS treatments

Sulfur volatile compounds are highly reactive chemical species, which have been shown to affect many organisms such as bacteria^42^, plants and fungi^43–45^. In terms of modes of action of these sVOCs, plants were shown to take up DMDS emitted by bacteria and use it as sulfur source^43^. Whether fungi or oomycetes are also capable of integrating sVOCs into their sulfur metabolism, or to which extent this metabolism would be affected by exposure to different sVOCs, is however so far unknown. To investigate this question, we first drafted a scheme of two main sulfur metabolism pathways^46^ in *P. infestans*, i.e. sulfate reduction and synthesis of cysteine/methionine. Since these pathways have not yet been studied in *P. infestans*, this scheme was based on the KEGG and Uniprot databases, and we used our proteomic data to complement the information.

**Figure 7:**
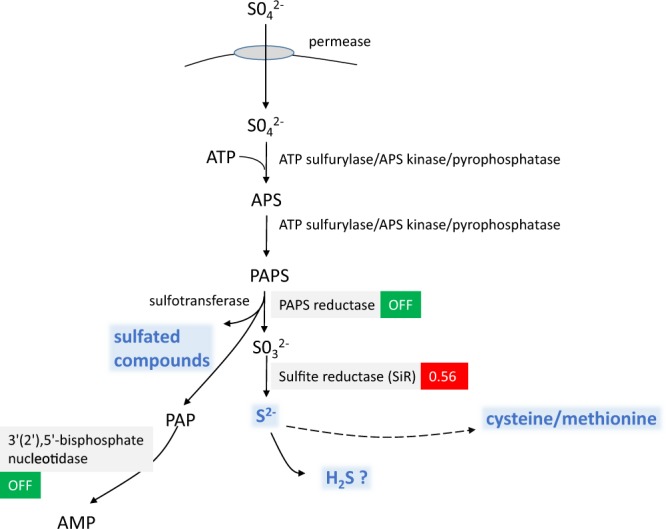
Theoretical schematic view of sulfur reduction in *Phytophthora* and effects of DMTS (red) and MMTS (green). The scheme was designed based on the presence in the proteome of *P. infestans* T30-4 of homologues of actors found classically in plants and fungi (adapted from^47^). Sulfate ions are transported by permeases across the plasma membrane and transferred in the cytoplasm to adenosine (ATP) by a tripartite enzyme comprising an ATP sulfurylase domain, a kinase domain (adenosine 5′-phosphosulfate kinase, APS kinase) and a pyrophosphatase domain^27^. The product of this reaction, the phospho-adenosine phosphosulfate (PAPS), is then converted to sulfite by the PAPS reductase, then into sulfide by the sulfite reductase (SiR). Sulfide can oxidize into hydrogen sulfide (H_**2**_S) or integrate the metabolism of cysteine and methionine. In plants, sulfation occurs on secondary metabolites or hormones, like glucosinolates and phytosulfokines and is controlled by the PAPS reductase and a sulfotransferase^48^. During this process, a harmful compound, called 5′-phosphoadenosine 3′-phosphate (PAP), accumulates. PAP is detoxified in plants by a 3′(2′),5′-bisphosphate nucleotidase. This enzyme was detected in the proteome of *P. infestans*. The mechanisms of sulfation in *Phytophthora* are unknown. Modifications in protein amounts observed upon sVOC treatments are shown in red (DMTS) and green (MMTS).

Homologues of genes encoding most enzymes involved in sulfate reduction in fungi^47^ and plants^46^ (ATP sulfurylase, APS kinase and PAPS reductase) were identified in the *P. infestans* genome (Fig. 7), although some differences might exist in oomycetes^27^. The capacity of *P. infestans* to reduce plant sulfate is crucial for infection, because the low methionine and cysteine levels in the apoplast are likely insufficient to sustain oomycete growth. Interestingly, two central sulfur reduction enzymes were less abundant upon MMTS treatment (Fig. 7): the PAPS reductase (D0N1L8), which reduces sulfate into sulfite; and the 3′(2′),5′-bisphosphatenucleotidase (D0N678), which – in plants – detoxifies 5′-phosphoadenosine 3′-phosphate (PAP) produced during sulfation of compounds by the PAPS reductase^48^. Moreover, DMTS reduced the amount of the sulfite reductase β subunit, involved in the final reduction of sulfite into sulfide (Fig. 7). The fold reduction of 0.56 was not statistically significant (Supplementary Table 1) but the decrease is worth mentioning. These first observations indicated that sulfur reduction, the first step in sulfur acquisition, might be affected in *P. infestans* mycelium exposed to MMTS and DMTS.

**Figure 8:**
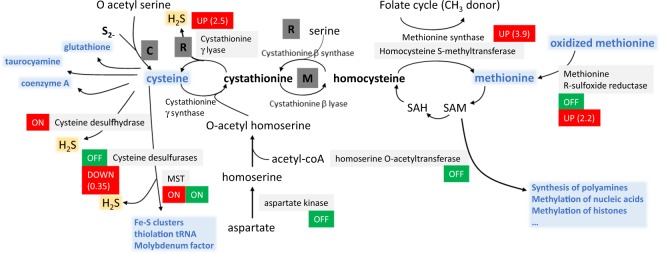
Theoretical schematic view of the synthesis of methionine and cysteine in *Phytophthora* and effects of DMTS (red) and MMTS (green). In plants, sulfide is incorporated by the cysteine synthase into O-acetylserine to produce cysteine (**C** = cysteine pathway). In fungi, the main pathway involves the synthesis of cystathionine from O-acetylhomoserine as substrate for the homoserine O-acetyltransferase. From cystathionine, the activity of the cystathionine γ lyase in the reverse trans-sulfuration pathway (**R**) results in cysteine production. The methionine synthesis pathway (**M**) involves the γ cystathionine synthase and the cystathionine β lyase, which synthetize homocysteine, the precursor for methionine. Methionine can be synthetized from homocysteine by the methionine synthase using folate as methyl donor or by the homocysteine S-methyltransferase using homocysteine and S-adenosyl methionine (SAM). All reactions depicted in this schema are controlled by enzymes annotated in the KEGG for *P. infestans*. The thiosulfate pathway does not seem to exist in *Phytophthora*. Also highlighted is the significance of cysteine as substrate for cysteine desulfurases to produce important cofactors (molybdenum cofactor, Fe-S clusters) and to thiolate tRNA (MST = mercaptopyruvate sulfurtransferase). Glutathione, coenzyme A, taurocyamine are also important sulfur compound produced from cysteine in *Phytophthora*. Methionine, besides being an important amino acid for proteins, is metabolized to S-adenosyl methionine (SAM), which participates in the methylation of nucleic acids or histones, or the synthesis of polyamines. Modifications in protein amounts observed upon sVOC treatments are shown in red (DMTS) and green (MMTS).

After reduction, sulfide is normally integrated into amino acids to form methionine and cysteine (Fig. 8). We identified several candidates controlling the synthesis of cysteine/homocysteine/methionine, which were differentially regulated by DMTS and MMTS (Supplementary Table 1; recapitulated in Fig. 8 and Table 2). First, DMTS led to higher abundance of enzymes involved in the synthesis of both cysteine (D0NAM5, 2.5x) and methionine (D0NMR7, 3.9x). Both amino acids are not only protein constituents, but also precursors of important sulfur-compounds (cysteine), such as glutathione, coenzyme A or Fe-S clusters, or methyl group donors (methionine) such as S-adenosyl methionine (SAM) used for DNA or histone methylation. DMTS led to lower levels of a cysteine desulfurase (D0NRJ8), which synthetizes cofactors involved in respiration such as Fe-S clusters^49^ or the molybdenum cofactor essential for the sulfite oxidase activity^50^. Desulfurases are also part of the sulfur relay system governing the thiolation of tRNA^51^. Thus, the lower content in cysteine desulfurase caused by DMTS might inhibit several biological processes essential for cell survival. In addition, upon treatment with DMTS or DMDS, we could detect a protein (D0NSN9) with homology to a L-cysteine desulfhydrase from *Arabidopsis lyrata* (XP_020879589.1; BlastP: 27% identity, 45% positive, with 81% coverage), which was absent in control samples. Cysteine desulfhydrase regulates the homeostasis of cysteine, which can be toxic at high doses in plants^52,53^ and algae^54^. This reaction generates H_2_S, which is also a by-product of both DMTS-upregulated enzymes mentioned above (cysteine desulfhydrase, cystathionine γ lyase)^55^. Therefore, it is tempting to speculate that DMTS exposure leads to accumulation of H_2_S in *P. infestans*. While the effect of H_2_S on oomycetes has not yet been reported, high doses of this compound are known to be toxic to plants and might also interfere with the oomycete biology. Further supporting a role of H_2_S in the sVOC-induced *P. infestans* inhibition, a protein (D0P2H7) with partial homology to mercaptopyruvate sulfurtransferase (MST) was detected in all sVOC-exposed samples but not in controls (Table 2). MST is involved in persulfidation of proteins, glutathione and cysteine in animal cells^56^, and regulates the thiolation of tRNA together with cysteine sulfhydrase. During these reactions, MST also produces H_2_S and other polysulfides. Moreover, another sulfide extracted from garlic, diallyl trisulfide (DATS), was also reported to induce the activity of MST in animal cells^57^. DATS is anti-carcinogenic and this recent study indicated that it might act as sulfur donor for the persulfidation of the Bcl2 protein by MST, which correlated with the inhibition of cell proliferation regulated by Bcl2^57^. Similarly, bacterial sVOCs might provide the sulfur for the oomycete MST, inducing its stability and therefore its higher abundance in sVOC-treated mycelium. The consequences of increased levels of MST on cell viability are unclear, since MST was more abundant in all sVOC-treated *P. infestans* independently of their inhibitory activity. While higher MST abundance is therefore unlikely responsible for the toxicity of DMTS and MMTS, it could be a general target of sVOCs and its effect might be additive to other defects caused by the individual sVOCs, e.g. H_2_S production upon DMTS treatment. A last interesting observation from the DMTS-induced changes in *P. infestans* proteome was the increased methionine R-sulfoxide reductase B (MsrB; D0MUU0), an enzyme involved in reverting the ROS-induced oxidation of thiomethyl groups on protein surface methionine^58^, thereby contributing to protein maintenance and cell survival. This observation suggests that DMTS treatment induced an oxidative stress in *P. infestans*, which is corroborated by the regulation of several other oxidative stress markers (Table 3), as discussed below.

**Table 2:** Proteins regulated by sVOCs and involved in the sulfur metabolism.

sVOC	Specificity	ID	Fold change	Molecular function (KEGG EC)	Biological process
MMTS		D0NMJ9 PITG_13686	ON	Unknown	Unknown, involved in C*hlamydomonas* S deficiency response^87^
		D0N1L8 PITG_04601	OFF	PAPS reductase *(EC:1.8.4.8?)*	Sulfur reduction (sulfation of compounds)
	*OFF with S-methyl*	D0N678 PITG_06011	OFF	3′(2′),5′-bisphosphate nucleotidase EC: 3.1.3.7	Detoxification of PAP after sulfation by the PAPS reductase
		D0P4C9; D0NSL6 PITG_21619/ PITG_15993	OFF	Homoserine O-acetyltransferase *(EC :2.3.1.31?)*	Synthesis of cystathionine
		D0NKQ0 PITG_12514	OFF	Aspartate kinase EC: 1.1.1.3 EC: 2.7.2.4	Synthesis of homoserine (cysteine cycle)
		D0NLG7 PITG_13223	OFF	Cysteine desulfurase *(EC:2.8.1.7?)*	Production of cofactors, Fe/S cluster, thiolation of tRNA
	*Induced with DMTS*	D0MUU0 PITG_01172	OFF	Methionine sulfoxide R reductase *(EC: 1.8.4.14?)*	Reduction of oxidized methionine
DMTS		D0NX78 PITG_18187	0.565	Sulfite reductase NADPH subunit β EC: 1.8.1.2	Sulfur reduction
		D0NAM5 PITG_08445	2.516	Cystathionine γ lyase EC:4.4.1.1	Cysteine synthesis
		D0NMR7 PITG_13769	3.89	Homocysteine S-methyltransferase *(EC:2.1.1.10?)*	Methionine synthesis
	*ON with DMDS*	D0NSN9 PITG_16020	ON	Putative desulfhydrase? *(EC:4.4.1.28?)*	Degradation of cysteine, production of H_2_S
		D0NRJ8 PITG_15057	0.354	Cysteine desulfurase NFS1 EC:2.8.1.7	Production of cofactors, Fe/S cluster, thiolation of tRNA
	*OFF with MMTS*	D0MUU0 PITG_01172	2.2	Methionine sulfoxide R reductase	Reduction of oxidized methionine
All VOCs		D0P2H7 or D0NYM9 PITG_20657 or PITG_18584	ON	Mercaptopyruvate sulfurtransferase (EC: 2.8.1.2*?)	Regulation of protein persulfidation and H_2_S production

EC numbers in italics and brackets are propositions of enzymatic functions. *Works with EC 2.8.1.7 in the sulfur relay system (thiolation of tRNA/synthesis of molybdenum) with production of H_2_S.

**Table 3:** Proteins regulated by DMTS or MMTS with putative function in response to oxidative stress.

sVOC	Specificity	ID	Fold change	Molecular function	Biological process
DMTS	*OFF by MMTS*	D0MUU0 PITG_01172	2.2	Methionine-R-sulfoxide reductase (*EC: 1.8.4.14?*)	Reduction of oxidized methionine
		D0NST2 PITG_16069	4.3	Chaperone HSP104	Refolding of disaggregated proteins
	*SM F* = *2.298* *BMTMS F* = *1.982*	D0NJX9 PITG_12948	1.969	Thioredoxin/protein disulfide isomerase	Cell redox homeostasis
		D0MRE5 PITG_00674	2.42	Carbonic anhydrase	Protection against oxidative stress
	*ON by MMTS*	D0NKS8 PITG_12541	ON	Selenoprotein T	Anti-oxidant
MMTS	*ON by DMTS* *BMTMS F* = *2.05* *(DMTS F* = *1.79* *SM F* = *1.82)*	D0MUU0 PITG_01172 D0NRD5 PITG_15492	OFF 2.034	Methionine-R-sulfoxide reductase *(EC: 1.8.4.14?)* Alkyl hydroperoxide reductase, thiol specific antioxidant = Peroxiredoxin 2 = thioredoxin peroxidase (uses thioredoxin)	Reduction of oxidized methionine Cell redox homeostasis: protects against reactive sulfur species oxidizing thiols
	*SM F* = *4.1*	D0N359* PITG_05579	2.957	Catalase	ROS detoxification
		D0NVL8 PITG_17249	0.40	Glutaredoxin (if reduced, higher ROS effect)	Redox regulation

*Or D0N358 (PITG_05578) not clearly identified.

In contrast to DMTS, MMTS reduced the abundance of proteins involved in the cysteine/methionine metabolism (Fig. 8). Although this might be due to the high toxicity of MMTS leading to general protein downregulation, it is worth noting that two enzymes involved in the production of homocysteine, the aspartate kinase (D0NKQ0) and the homoserine O-acetyltransferase (D0P4C9) were not detectable in the MMTS-treated samples (Fig. 8, Table 2). Likewise, the methionine sulfoxide reductase (D0MUU0), which was more abundant in the DMTS-treated samples, could not be detected either, nor could another isoform of cysteine desulfurase (D0NLG7). Thus MMTS led to decreased abundance of important sulfur metabolism enzymes in *P. infestans*.

Altogether, our data suggest that exposure to DMTS and MMTS (but not to the three other, less active sVOCs) creates an imbalance in the sulfur metabolism of *P. infestans*, which might partially explain the strong anti-oomycete activity of these two sVOCs. Interestingly, two fungicides (pyrimethanil and cyprodinil) were proposed to target the methionine synthesis pathway in fungi^59^ (although this was recently revisited for the effect of pyrimethanil on *Botrytis*^60^). Moreover, methionine synthesis was recently shown to be essential for virulence in the rice blast fungus *Magnaporthe grisea*^61^. In *P. infestans*, the methionine synthase transcripts accumulated in the appressorium during infection and methionine concentration was shown to vary during cyst germination and appressorium formation^62^. Similarly, a proteomic approach revealed that *in vitro* germinated cysts and appressoria of *P. infestans* displayed a higher content in two isoforms of the methionine synthase, when compared to the mycelium^63^, suggesting that methionine is important for oomycete virulence. There is less information on the role of cysteine in the biology of *P. infestans*. However, many cysteine-rich proteins are encoded in the *P. infestans* proteome. These proteins, which are likely secreted, have been proposed to be virulence factors or toxins^64–66^. For example, the cysteine-rich protein SCR96 was shown to determine pathogen virulence and oxidative stress tolerance in *P. cactorum*^67^ and other types of cysteine-rich proteins are likely to play important roles in *P. infestans* infection^68^. Thus an impairment of the cysteine metabolism might not only impact the oomycete growth but also affect its ability to infect plant tissues, which is of particular interest for crop protection.

### Additional changes in the proteome caused by MMTS

Beside sulfur metabolism, MMTS affected many other important biological processes, as judged by the large number of regulated proteins in all functional categories (Supplementary Table 1). The 5 times higher abundance of D0NCV5, a putative Pleiotropic Drug Resistance protein (PDR1-15) from the ABC superfamily, supports the high toxicity of MMTS for the oomycete. PDR proteins function as efflux pumps to dispose of xenobiotics^69^. So far little is known about the way oomycetes detoxify xenobiotics: their genomes encode less cytochromes P450 than ascomycetes, but more ABC transporters^27,69^. Therefore, the upregulation of D0NCV5 might represent an attempt to detoxify MMTS and this transporter might constitute a target to consider when investigating the role of ABC transporters in the sensitivity or resistance of *P. infestans* to fungicides or antimicrobials^70^. One additional defence mechanism seems to be activated in MMTS-treated *P. infestans*, as indicated by the enrichment of a catalase and a peroxiredoxin-2, both involved in redox homeostasis (Table 3). Beyond MMTS, these redox changes seemed to occur upon exposure to all sVOCs, which led to an increased abundance of different antioxidants compared with the control (Table 3; Supplementary Table 1).

While sulfides (DMDS, DMTS), might participate in sulfhydration of free thiols of cysteine residues, the sulfonate MMTS is certainly more potent to modify thiol groups. It is actually used in chemistry to study thiol modifications on proteins^71^, since it alkylates reversibly the free thiol groups present on cysteine residues. This might explain why MMTS led to more changes in the proteome pattern of *P. infestans* compared to the other tested sVOCs. The high reactivity of MMTS is due to the presence of oxygen atoms near the sulfur center, as described for the related sulfur compound allicin^72^. Allicin (diallyl thiosulfinate) is another thiol modifier, which has been extensively studied in biological research because of its health-promoting properties^73^. This volatile is very abundant in *Allioidae*, including garlic, and is a precursor for sulfinates, such as diallyl trisulfide (DATS), whose role in protein persulfidation was mentioned above. Allicin has long been known as potent inhibitor of many microbes^72,74^, including *Phytophthora*^75,76^. It is also widely studied for its anticancer properties in the medical field and has a long list of cellular targets^73^. As for allicin, the antimicrobial activity of MMTS might lie in its high reactivity to the protein thiols and our proteome data also suggest multiple cellular targets in *P. infestans*. More advanced approaches, e.g. analysing specifically the S-thioallylation of proteins as recently performed for allicin^77^, should help to better understand the effects of MMTS and DMTS on *P. infestans* physiology and cellular biology, which underlie the strong anti-oomycete activity of these two sVOCs.

## Conclusions and perspectives

Prior to this work, we hypothesized that bacteria naturally associated with plants might be a source for novel antimicrobial compounds^14^. We therefore characterized the volatiles emitted by beneficial potato-associated *Pseudomonas* strains and observed that several sVOCs inhibited the growth of the late blight causing agent *P. infestans*^19^. Here, a more comprehensive investigation of the activities of these sVOCs was conducted on both the plant and the pathogen, which showed that:

- The trisulfide DMTS and the thiosulfonate MMTS were both capable to prevent late blight on leaf discs and potato plantlets, but only MMTS did so without inducing phytotoxicity. In agronomy, only DMDS has been used so far for field application to control nematodes, various soil-borne plant disease and weeds^24,78^. However, our study showed that MMTS was a much better protectant of leaf discs and plantlets against late blight than DMDS.
- MMTS was inactive as pre-treatment and did not induce plant defences. Our data indicated that MMTS acted through direct anti-oomycete activity.
- Different sVOCs induced specific changes in the proteome of *P. infestans*. BMTMS affected the translational machinery and DMTS perturbed many important steps of sulfur metabolism. MMTS also perturbed sulfur metabolism along with many other cellular processes, including redox balance, suggesting multi-target modes of action for this potent anti-oomycete volatile compound.

We propose that MMTS, a thiosulfonate volatile compound produced by both plants and bacteria, plays an important role in plant defence against pathogens. Although MMTS was not phytotoxic in our experimental setup, future studies shall investigate the putative toxicity of this sVOC towards non-target organisms to evaluate its suitability for crop protection. Beyond this translational research aspect, our study raises many fundamental questions related to (i) the molecular targets of sVOCs in inhibited organisms such as *P. infestans*, (ii) the molecular determinants underlying sVOC synthesis and regulation in plant-associated bacteria, and (iii) the primary function of sVOCs for bacterial physiology and plant-bacterial interaction. Rather than being recognized as MAMPs, MMTS and DMTS dampened the plant response to flagellin. Therefore, MMTS and other sVOCs might first act as effectors to allow the initiation of the beneficial association, before contributing to the host defence against pathogens to ensure the sustainability of this association.

## Material and Method

### Plant material and growth conditions

Potato tubers of the cultivar Bintje were potted and grown for four to six weeks (photoperiod 18 h, relative humidity 60 to 70%, 25/20 °C during the light/dark period). Leaf discs were sampled using a cork borer of 15 mm diameter from four to five individual plants using the fourth to the fifth or the fifth to the seventh leaves depending on the age of the plant. Leaf discs were incubated overnight on water agar plates (0.8% agar, LP0011, Oxoid) before infection with *P. infestans* and/ or treatment with sVOCs. Disease- free *in vitro* potato plantlets of the Victoria cultivar were provided by JP De Joffrey (Agroscope, Changins, Nyon). They were subcultured at 25 °C (18 h light; 23 °C at night) by cutting the shoot into three pieces below the axils and transferring into fresh medium. Plantlets of ten to fourteen days were used for the VOC and infection assays. The Arabidopsis plants (Columbia Col-0) used in the ROS assays were grown as one plant per pot at 21 °C with an eight-hour photoperiod for five weeks.

### Volatiles

Sulfur volatiles were ordered from Sigma: Dimethyl disulfide (DMDS, n° W353604), Dimethyl trisulfide (DMTS, n° W327506), S-methyl methane thiosulfonate (MMTS, n°64306), S-methyl butanethioate (n° 277819, SM) and bis(methylthiomethyl) sulfide (BMTMS, CDS000802). Dimethyl sulfoxide (DMSO) was used as solvent control.

### Phytophthora infestans *strain and culture*

*Phytophthora infestans* Rec01 collected at the Agroscope station of Reckenholz^5^ was used for all experiments. The isolate was maintained as mycelial culture on V8 medium supplemented with 15% agar (Agar-agar, Kobe I, Roth) and 0.1% calcium carbonate. Its virulence was preserved by regular passage on Bintje potato tubers. Petri dishes were incubated upside down in a plastic bag (no sealing) in the dark at 18 °C.

### *Volatile treatment on* P. infestans

The effect of sVOCs on mycelial growth of *P. infestans* was assessed using 5 mm agar plugs from the edge of actively growing mycelial colonies, which were placed downward-faced in one compartment of bi-plates (Sarstedt n°82.1195) filled with fresh V8 medium. Defined amounts of the sVOCs were applied pure or diluted in DMSO on a dry droplet of 100 μl of water agar (1%). Plates were sealed with Parafilm M and incubated upside-down in the dark at 18 °C. Mycelial growth was monitored seven days after inoculation by taking photographs and total mycelial area was further assessed using ImageJ.

### *Plant infection assays with* P. infestans

Zoospores were released from sporangia developed on two to three-week *P. infestans* plates using a treatment with ice-cold sterile water and further incubation for two hours in the fridge. After 20 min at room temperature spores were pipetted from the water surface and counted in a Jessen cell chamber. Infection assays on leaf discs were performed using a 100’000 zoospores.mL^−1^ by applying a ten- microliter droplet in the center of each leaf disc (abaxial surface). Petri dishes were incubated in a polystyrene box containing wet paper for six days at 18 °C in the dark. In this assay, volatiles were applied after dilution into DMSO as two-microliter droplets loaded on PTFE/silicone septa (8 mm; n° 507784 from Sigma) placed in the center of the Petri dish. For the infection of *in vitro* plantlets in sterile tubes (50 mL filled with 10 mL of medium), a plug of 1% water agar was loaded inside the lid where the sVOC or solvent was applied for further treatment as two-microliter droplet. One leaf of the plantlets was inoculated with ten microliters of *P. infestans* zoospores (100’000 spores.mL^−1^) and incubated at 18 °C under light. Pictures were taken six days post-infection to evaluate the spreading of late blight on the whole plantlets (Fig. S11). The weight of each plantlet shoot was measured after cutting the root system.

### Fatty acid methyl esters analysis (FAME)

FAMEs were prepared from control and *P. infestans* -inoculated potato leaf discs treated or not with sulfur volatiles, by acid-catalysed transesterification. Samples (3 leaf discs each) were incubated in 1 ml of 5% H_2_SO_4_ in MeOH (v/v), 50 μL of 0.05% butylated hydroxytoluene (w/v) in MeOH and 10 μg of glyceryl triheptadecanoate (Sigma Aldrich, Buchs, Switzerland) used as an internal standard. The reaction was carried out at 85 °C for 45 min in 7-ml glass tubes. Tubes were then cooled down at room temperature, briefly centrifuged, and 1.5 mL of 0.9% NaCl (w/v) and 2 mL of *n*-hexane were added. Samples were thoroughly shaken for 5 min and centrifuged at 240 *g* for 5 min. The upper organic phase containing the FAMEs was transferred into a new glass tube and the extraction was repeated two additional times with 2 mL *n*-hexane each time. The pooled organic phases were evaporated with nitrogen and resuspended into 200 μL of heptane. FAME samples (2 μL) were separated and quantified by GC-FID in split mode (50:1) equipped with a 30 m x 250 μm x 0.25 μm DB-23 capillary column (Agilent technologies) as previously described^79^. The chemical identification of the *P. infestans*-specific fatty acid 20:5 n-3 ((5*Z*,8*Z*,11*Z*,14*Z*,17*Z*)-5,8,11,14,17-eicosapentaenoic acid; EPA) was originally determined by co-migration with a 20:5 n-3 authentic standard (Supelco 37 component FAME mix; Sigma) by GC-FID analysis. It was further confirmed by GC-MS-EI analysis in splitless mode with the same capillary column and oven program as for GC-FID analysis. The injection port and detector temperatures of the GC-MS were set at 250 and 230 °C, respectively. Mass spectra were obtained by electron ionization set at 70 eV with a data acquisition rate of 50 Hz. The EPA mass spectrum was compared to 20:5 n-3 authentic standard (Supelco 37 component FAME mix) and with the EPA methyl ester reference mass spectrum (http://www.lipidhome.co.uk/ms/methesters/me-5plus/index.htm).

### Oxidative burst assays

The effect of sVOCs on the defence response of Arabidopsis was evaluated by measuring the production of reactive oxygen species (“oxidative burst”). Briefly, leaf discs were floated on water overnight and ROS released by the leaf tissue were measured using a luminol‐based chemiluminescent assay^34^. ROS were elicited with 1 μM flg22 peptide (QRLSTGSRINSAKDDAAGLQIA, obtained from EZBiolabs) in all experiments. Mock treatments without flg22 were performed with the control solution (1% w/v BSA, 100 mM NaCl) used to solubilize the peptide. sVOCs were applied at the indicated doses (0 to 10 μg) and DMSO was used as solvent control. Luminescence emitted by the oxidized L-012 luminol (Wako Chemicals USA) was measured over a time period of 30–35 min using a luminometer (Glomax, Promega, Switzerland).

### Q-PCR analysis of gene expression

The effect of sVOCs on defence gene expression was analysed by qPCR in 4-week old potato plants. Leaf discs from three plants were sampled and incubated on water agar plates at room temperature for 6 h. Leaves were then infected (or not) with zoospores of *P. infestans*, and co-treated (or not) with 1 mg of volatiles as described above. After 6 h of treatment, leaf discs were collected, snap-frozen in liquid nitrogen and stored at −80 °C. RNA were extracted from leaf discs using the phenol-chloroform extraction method using Trizol solution (38% (v/v) saturated phenol (pH 8), 0.8 M guanidine thiocyanate, 0.4 M ammonium thiocyanate, 0.1 M Na-acetate pH 5, 5% (v/v) glycerol). RNA extracts were treated with the DNase I from Sigma Aldrich and reverse transcription was performed using the SensiFAST cDNA Synthesis Kit from Bioline. Quantitative PCR reactions were performed using the SensiFAST SYBR Hi-ROX Kit from Bioline. Each reaction was carried out with 5 µL of cDNA (5 ng.µL^−1^), 7.5 µL of SYBR Hi-ROX mix, 0.5 µL of each primer and 1.5 µL of sterile water. For amplification, an initial denaturation step at 95 °C for 15 min was done, followed by 45 amplification cycles (95 °C for 15 s, 60 °C for 15 s, 72 °C for 30 s). Each reaction was run in duplicate and the experiment was repeated twice. All results were analysed using the double delta Cq method with uninfected samples treated with water as references. Primers for the defence genes *ERF3*, *LOX*, *PR-1b* and *PR-5* from potato^37^ were newly designed and are listed in Supplementary Table 2. Genes coding for the peptidyl-prolyl isomerase (CyP) and Elongation Factor α (EF1-α) were used as reference for normalization of data. A two-way ANOVA followed by Tukey-HSD post-hoc test was performed for statistical analysis with *p* ≤ 0.05.

### *Proteomic analysis of sVOC- treated* P. infestans *cultures*

*P. infestans* was grown on 10 mL Rye agar medium^19^ at 18 °C for ten days in one compartment of a 80 mL bi-plate. Thereafter, 300 μg sVOC (or DMSO as control) were pipetted on agar plugs in the other compartment of the Petri dish. This concentration was selected as that at which even the moderately active BMTMS reduced *P. infestans* mycelial growth (Fig. S10). Plates were sealed with parafilm, packed in a plastic bag and incubated upside down for 24 hours at 18 °C in the dark, after which *P. infestans* mycelium was collected by scratching the agar surface with a glass coverslip and frozen. Samples were frozen, ground in liquid nitrogen and stored at −80 °C before protein extraction. The protein extracts of the six different treatments from the three independent biological experiments (n = 18) were prepared concomitantly. To this end fifty milligrams of frozen powder were resuspended into cold SDS- extraction buffer (50 mM Tris HCl pH 7.5, 150 mM NaCl, 1% SDS) supplemented with antiproteases (Complete, Sigma).

Concentration of protein extracts was determined using Roti Nanoquant (Carl Roth). 30 µg of protein extract per sample were separated on 1D-SDS PAGE and stained with Blue silver colloidal Coomassie^80^. Lanes were cut into 15 equidistant pieces and subjected to tryptic in-gel digestion as described earlier^81^. Resulting peptide mixes were desalted using C18 Zip Tips (Millipore). LC-MS/MS analyses were done using an EASY-nLC, coupled to an Orbitrap Velos mass spectrometer (Thermo Scientific). Peptides were separated on in-house self-packed nano-LC columns (100 µm × 20 cm) containing reverse-phase C_18_ material (3.6 µm, Aeris, phenomenex) and eluted by a non-linear binary gradient of 77 minutes from 5% to 99% solvent B (0.1% acetic acid (v/v), 99.9% acetonitrile (v/v)) in solvent A at a constant flow rate of 300 nl min^−1^. Samples were measured in LTQ/Orbitrap parallel mode, survey scans in the Orbitrap were recorded with a resolution of 60,000 in a m/z range of 300–1,700 and the 20 most intense peaks were subjected to CID fragmentation in the LTQ. Dynamic exclusion (30 sec) of precursor ions was enabled, single-charged ions as well as ions with unknown charge-state were excluded from fragmentation. Internal lock-mass calibration (lock mass 445.120025) was enabled.

Database searching and quantification was performed using MaxQuant software v1.5.7.0^82^. MS and MS/MS spectra were searched against a *P. infestans* T30-4 database (uniprot version 2017-08-09; containing 17,612 entries) using following parameters: protease trypsin, two missed cleavages allowed, variable modification methionine oxidation, precursor ion mass tolerance 10 ppm, fragment ion mass tolerance 0.5 Da. Protein quantification was based on LFQ intensities based on at least two peptides. False discovery rate on peptide and protein level was set to a maximum of 1%. Mass spectrometry proteomics data have been deposited to the ProteomeXchange Consortium via the PRIDE partner repository^83^ with the data set identifier PXD014455.

For determination of statistically significant changes on protein level comparing control and treated biological replicate groups the Students T-Test was applied. For identifying the candidates regulated by specific sVOCs, the raw lists of sVOC- regulated proteins were manually examined (Supplementary Table 1, curated lists). The proteins listed as curated were used to produce the Venn diagrams presented in Fig. 6 and to calculate the percentage of regulated proteins (total number of unique proteins identified n = 3348). For those of special interest described in the main text, EuKaryotic Orthologous Groups (KOG) and Gene Ontology (GO) annotations were re-examined and an analysis using BLASTp against the non-redundant protein database was conducted to identify homologs in other species. The GO enrichment analysis was done using the DAVID Bioinformatics Resources 6.8 (https://david.ncifcrf.gov)^84,85^, while the pathway enrichment analysis was conducted using the Kyoto Encyclopaedia of Genes and Genomes (KEGG) Pathway database50. For treemap based data visualization proteins were assigned to KOG based functional categories. The treemap’s cells sizes were determined by the average protein occurrence level within all samples. Data analysis was performed by using Multi-Experiment Viewer^86^ and Treemap generation by using Paver (Decodon Greifswald).

## Supplementary information

Supplementary Information

Supplementary Information

Supplementary Information

Supplementary Information
