# Supplementary material for: A sulfur-containing volatile emitted by potato-associated bacteria confers protection against late blight through direct anti-oomycete activity
Source: Sci Rep. 2019 Dec 30;9:18778. doi: 10.1038/s41598-019-55218-3 (PMC6937334; doi:10.1038/s41598-019-55218-3)
Supplement: Supplementary file 1 — Supplementary Information [file 41598_2019_55218_MOESM1_ESM.docx]

**Supplementary information for «  A sulfur-containing volatile emitted by potato-associated bacteria confers protection against late blight through direct anti-oomycete activity” by Chinchilla et al.**

Delphine Chinchilla, Sébastien Bruisson, Silvan Meyer, Daniela Zühlke, Claudia Hirschfeld, Charlotte Joller, Floriane L’Haridon, Laurent Mène-Saffrané, Katharina Riedel, Laure Weisskopf

Supplementary table 1: proteomic data (xcel file)

Supplementary table 2: qPCR primers (xcel file)

Supplementary data (pdf presentation): Voronoi treemaps

Supplementary figures S1-S10


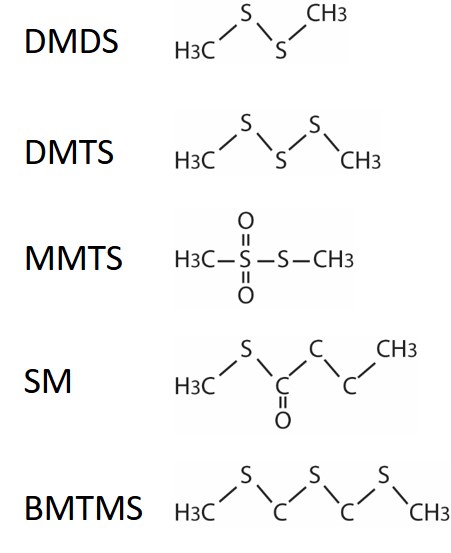


**Figure S1. Chemical structures of the different sVOCs studied.** DMDS: dimethyl disulfide; DMTS: dimethyl trisulfide; MMTS: S-methyl methane thiosulfonate; SM: S-methyl butanethioate, BMTMS: bis(methylthiomethyl) sulfide

**
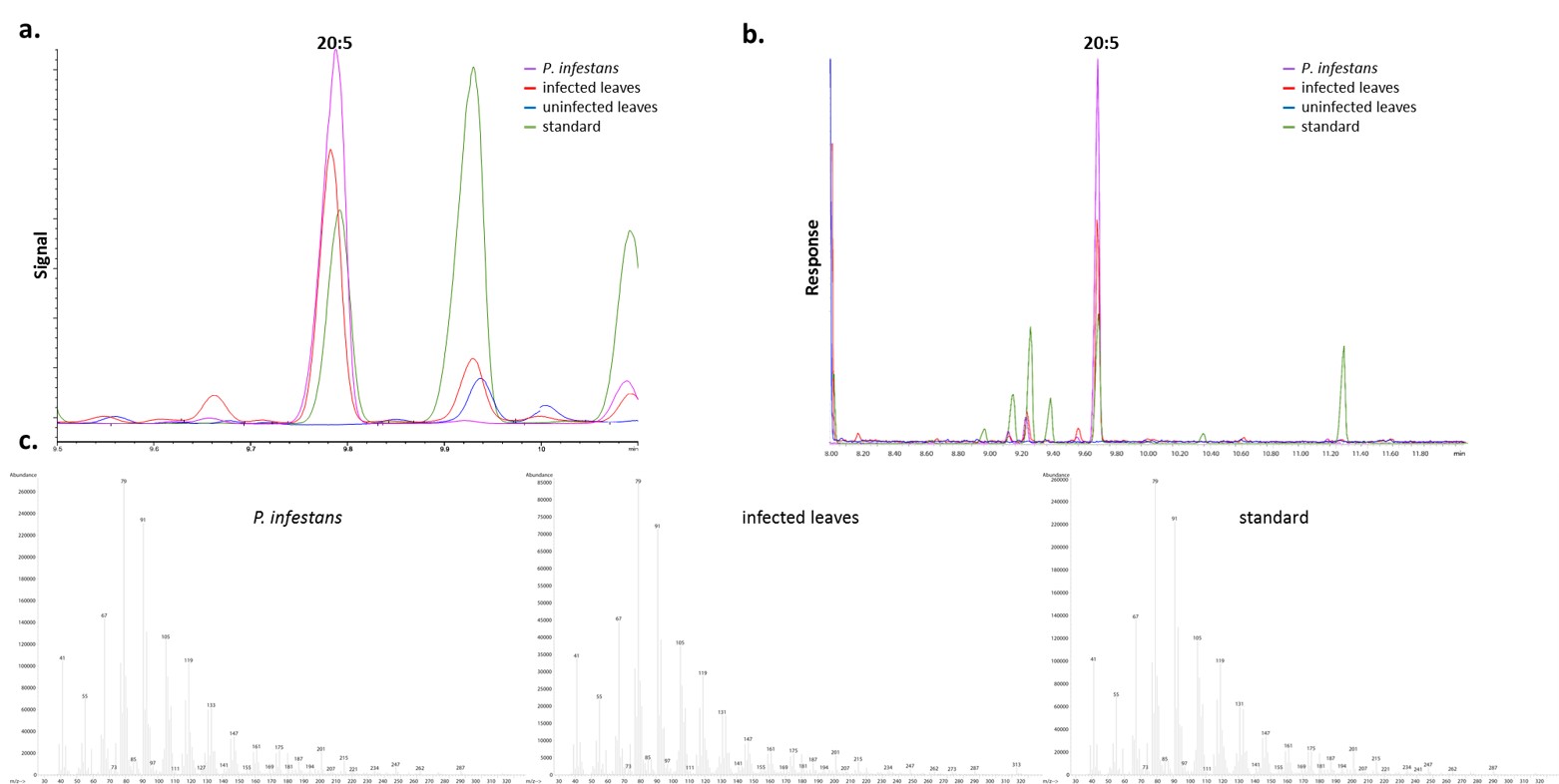
**

**Figure S2. Fatty acid marker Eicosapentaenoic acid (20:5 n-3) of *P. infestans* in late blight-infected potato leaves 6 days after infection**. a. GC-FID chromatograms of pure culture of *P. infestans* (purple), of leaves infected for 6 days with *P. infestans* (red), of leaves non infected (blue) and pure standard (Supelco 37 component FAME mix, Sigma). b. GC/MS spectra for the peak of interest. c. 20:5 mass spectra for the indicated samples.


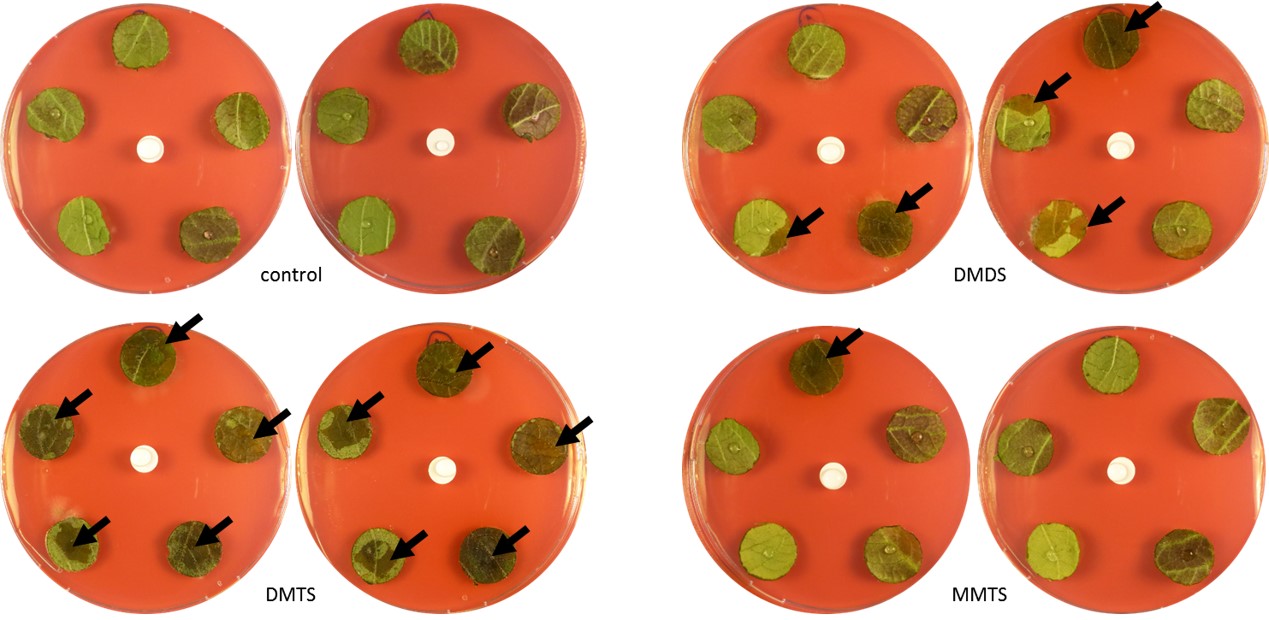


**Figure S3. Phenotype of leaf discs treated with sVOCs only.** Leaf discs of five different plants were treated for 6 days with 1 mg of DMDS, DMTS or MMTS (n=2 replicates). The difference in colour shown by leaf discs in the control treatment is likely due to different anthocyanin contents and is unrelated to the treatment performed (solvent or sVOCs). Note the water soaking symptoms in DMDS- and DMTS- exposed samples marked by black arrows, less frequently observed in the MMTS-treated samples.


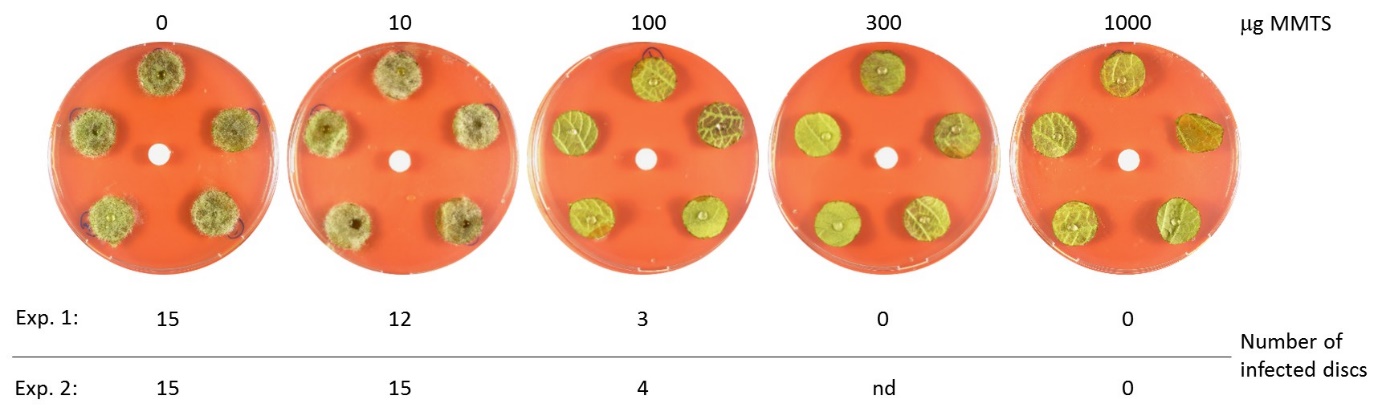


**Figure S4. MMTS protects potato leaf discs against late blight in a dose-dependent manner.** Leaves were treated with increasing doses of MMTS concomitantly with the pathogen inoculation. Pictures are representative of two independent experiments and the number of leaves out of 15 exhibiting a mycelial mat after inoculation is indicated below.


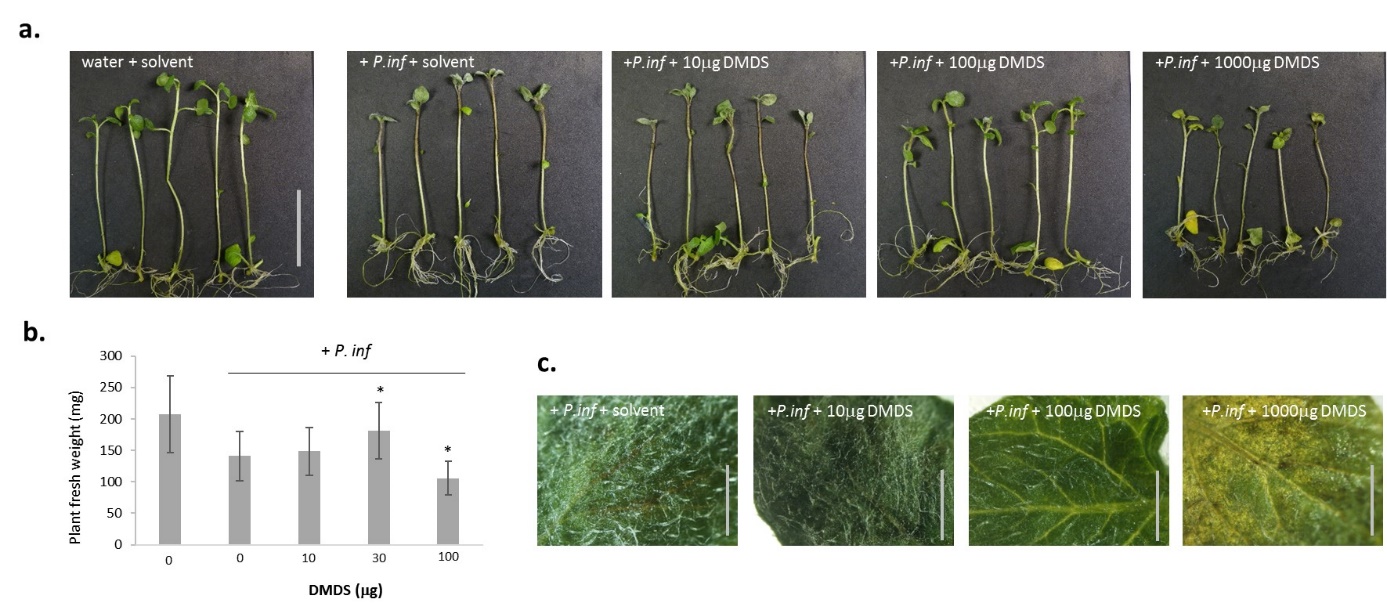


**Figure S5. DMDS treatment on potato plantlets inoculated with *P. infestans*.** a. Phenotype of Victoria plantlets six days of incubation with (or without) *P. infestans* (*P. inf)* exposed to 0, 10, 100 and 1000 mg DMDS. Scale bar = 5 cm. **b.** Quantification of the fresh weight of the respective plantlets. The bars show averages of ten individual plantlets with error bars indicating standard deviation. Signiﬁcant differences to the control treatment (=“0”) according to an ANOVA test are marked by asterisk: *, p <0.05. **c.** Binocular pictures at the leaf surface. Scale bars = 3 mm.


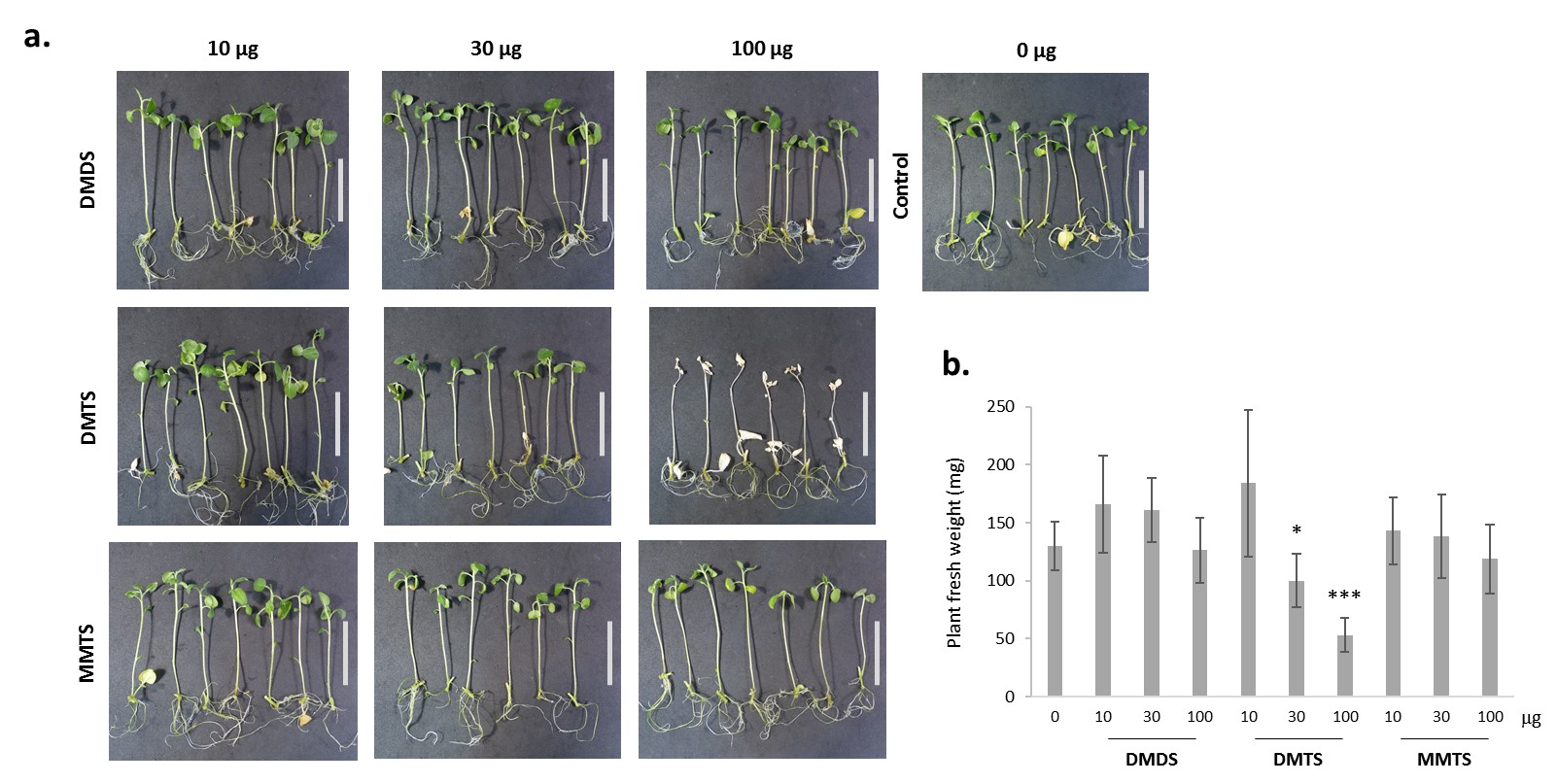


**Figure S6. Effect of sVOCs on plantlet development in the absence of *P. infestans*.** a. *In vitro* grown potato plantlets (cv Victoria) were exposed to sVOCs (top to bottom: DMDS, DMTS, MMTS) at 10, 30 and 100mg (left to right). Control treated with solvent is shown on the right. Scale bars = 5 cm. b. Fresh weight was measured for seven plantlets. The bars show averages with error bars indicating standard deviation. Signiﬁcant differences to the solvent control (=“0”) according to an ANOVA test are marked by asterisks: *, p <0.05; and ***p<0.001.


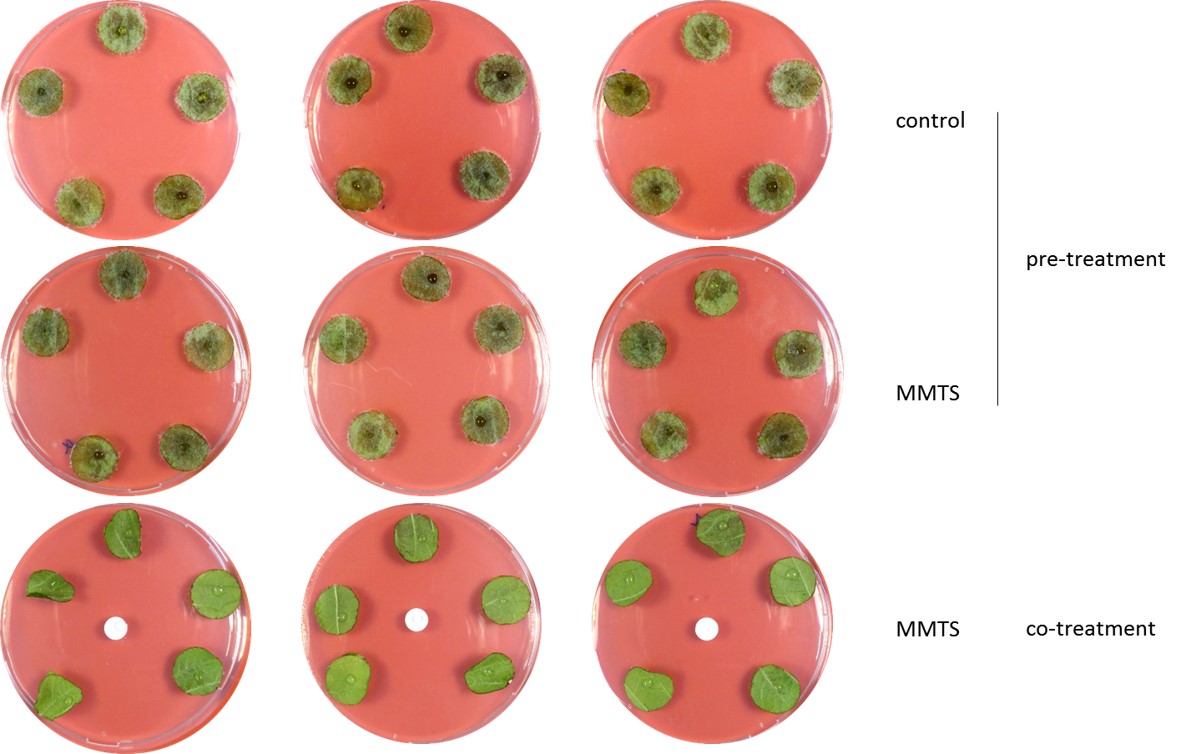


**Figure S7. Pre-treatment with MMTS is not sufficient to protect potato leaf discs against late blight**. A 24h-treatment with 1mg MMTS was applied to leaf discs in a volatile assay. Discs were transferred on fresh agar plates and six hours later, inoculated with *P. infestans* as describe before. Symptoms exhibited by MMTS-pre-treated leaf discs (middle) were as strong as in the solvent controls (bottom), while abolished by the MMTS (1mg) supplied as co-treatment (bottom).


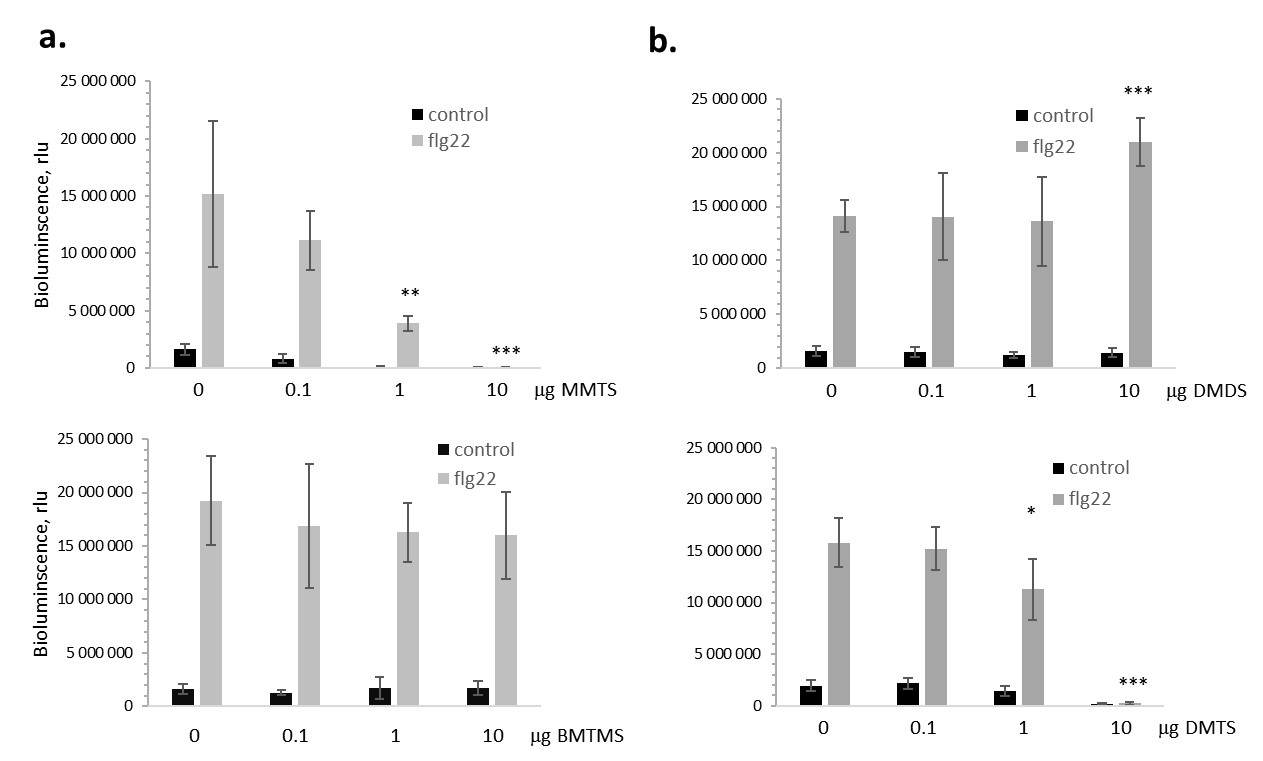


**Figure S8. The effect of sVOCs on flg22-induced oxidative burst.** Dose-response of sVOCs on the oxidative burst induced by flg22 in Arabidopsis. The bars show averages of six replicates with error bars indicating standard deviation. Signiﬁcant differences according to an ANOVA test are marked by asterisks: *, p<0.05; **, p<0.001 and ***p<0.001. Note that the increase in luminescence signal upon flg22 and DMDS treatment (marked by ***) was found in only one of the two assays performed.


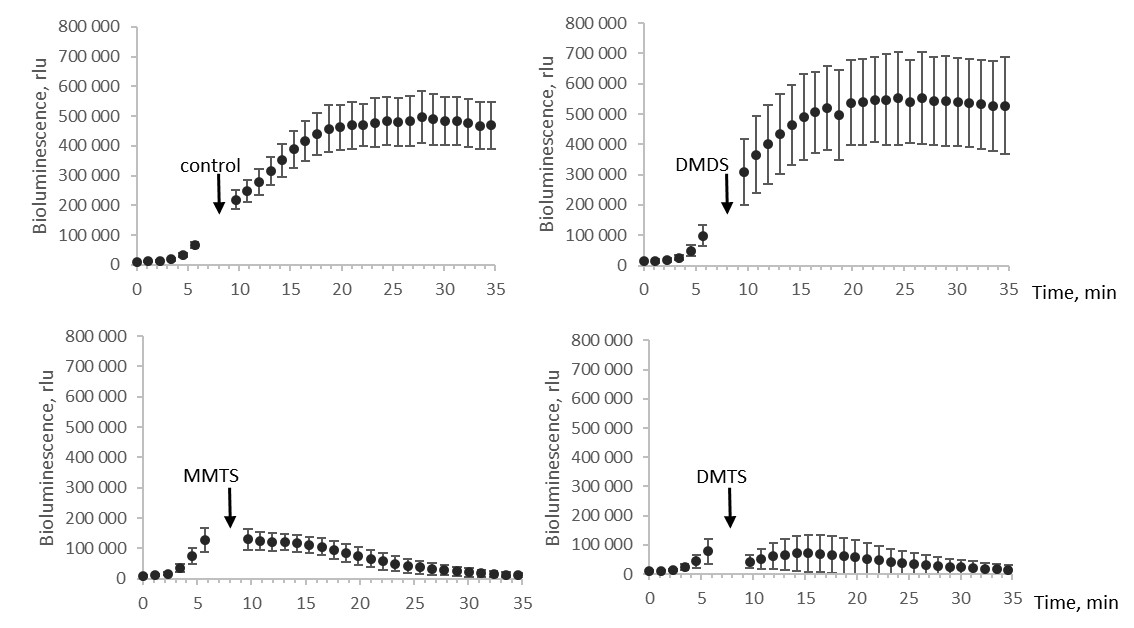


**Figure S9. The effect of sVOCs on flg22-induced oxidative burst.** Arabidopsis leaf discs were first elicited with 1mM flg22, the response was evaluated, and a treatment with 10mg sVOC was applied at ̴ 8 min after elicitation with flg22 (arrow). The bars show averages of six replicates with error bars indicating standard deviation.


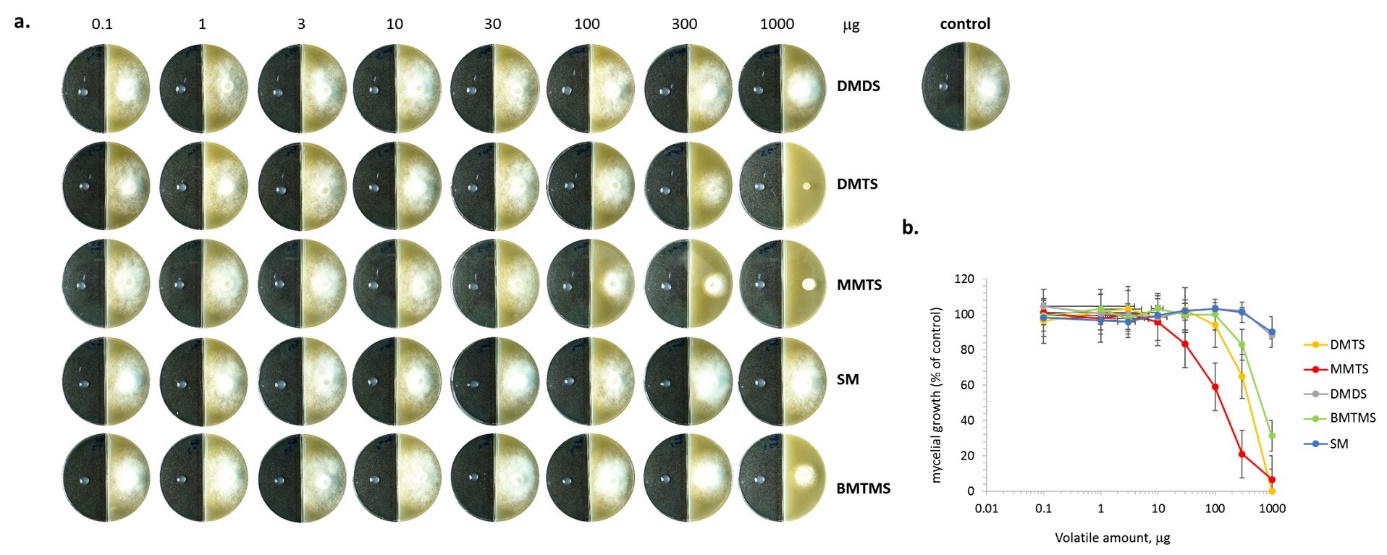


**Figure S10. The effect of five sVOCs on radial growth of *P. infestans*.** a. Plugs of *P. infestans* were incubated in the first compartment of bi-plates containing V8 solid medium and the volatile of interest was loaded on an agar plug in the second compartment. Pictures shown were captured after 7 days of incubation at room temperature and are representative of n= 3 -7 replicates. b. Quantification of the radial hyphal growth was performed using ImageJ. The results obtained with the volatiles were normalized to the ones obtained with control samples (100%) and presented as percentage of radial growth.


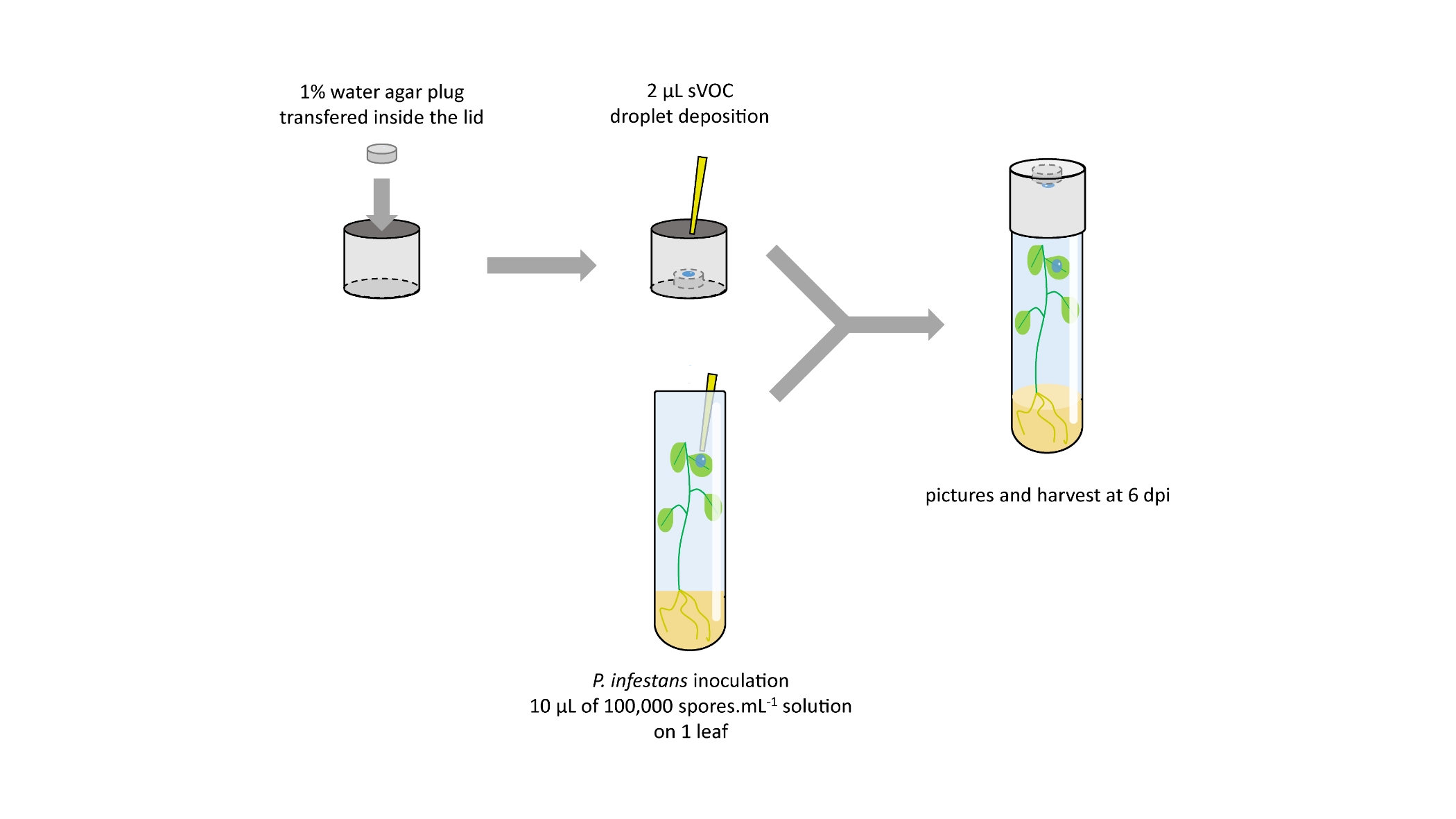


**Figure S11. Experimental procedure for *in vitro* plantlet treatment and infection***. In vitro* plantlet were treated with 2 µL of sVOCs applied on a 1% water agar plug loaded inside the lid. One leaf per plant was infected using 10 µL of a 100,000 zoospores.mL^-1^ solution.
