# Supplementary material for: A sulfur-containing volatile emitted by potato-associated bacteria confers protection against late blight through direct anti-oomycete activity
Source: Sci Rep. 2019 Dec 30;9:18778. doi: 10.1038/s41598-019-55218-3 (PMC6937334; doi:10.1038/s41598-019-55218-3)
Supplement: Supplementary file 2 — Supplementary Information [file 41598_2019_55218_MOESM2_ESM.pdf]

Effect of **DMDS** exposure on protein pattern of *Phytophthora infestans*

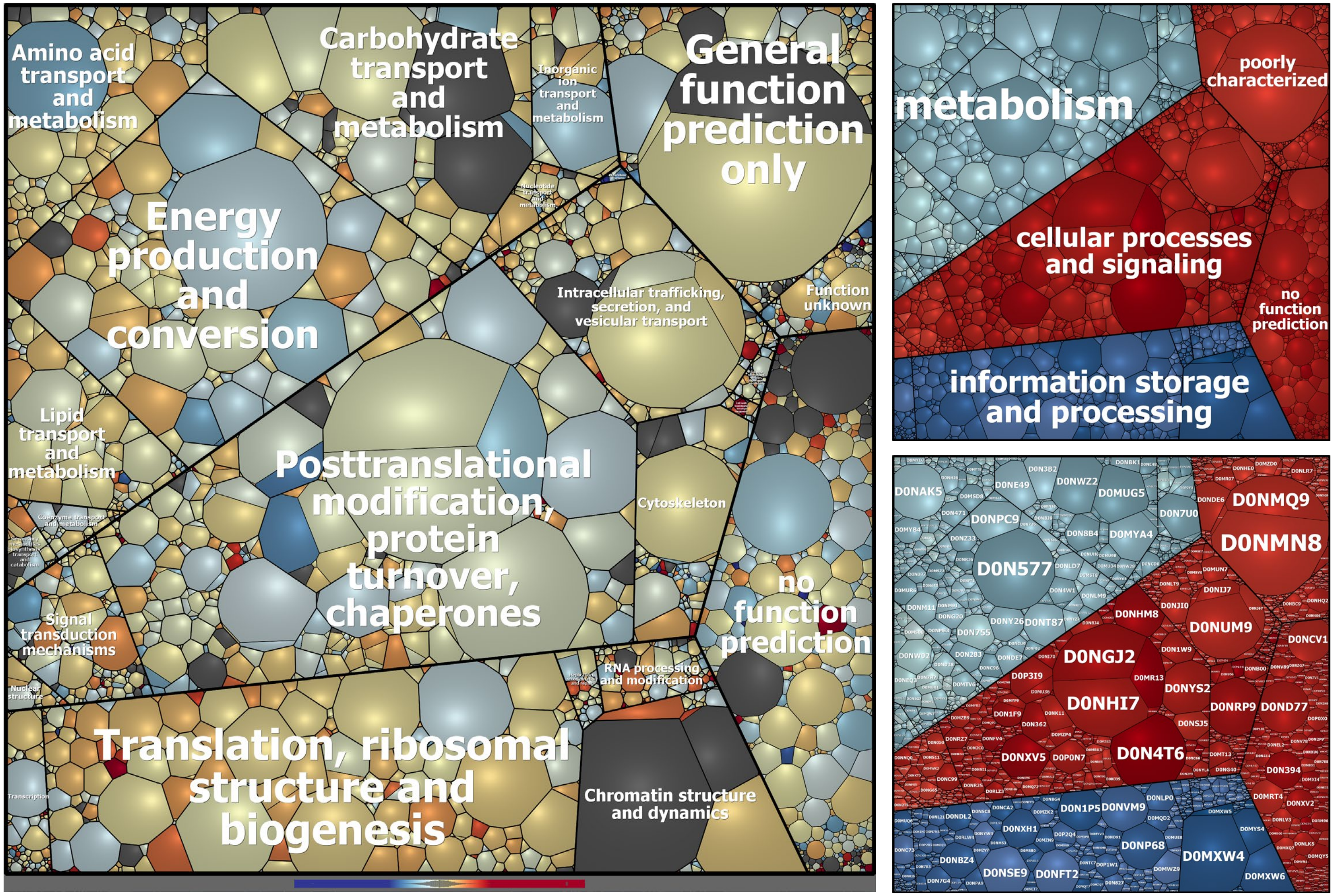

Voronoi treemap visualization of protein expression pattern of *P. infestans* exposed to DMDS.
Relative changes compared to untreated control are color-coded: red – higher amount in treated, blue – higher amount in untreated cultures. Cells sizes were determined by the average protein occurence level within all samples. Proteins are represented by cells, cells are clustered according their functional classification. Classification was done using Prophane 2.0. Grey cells indicate proteins that were not identified.

Effect of **DMTS** exposure on protein pattern of *Phytophthora infestans*

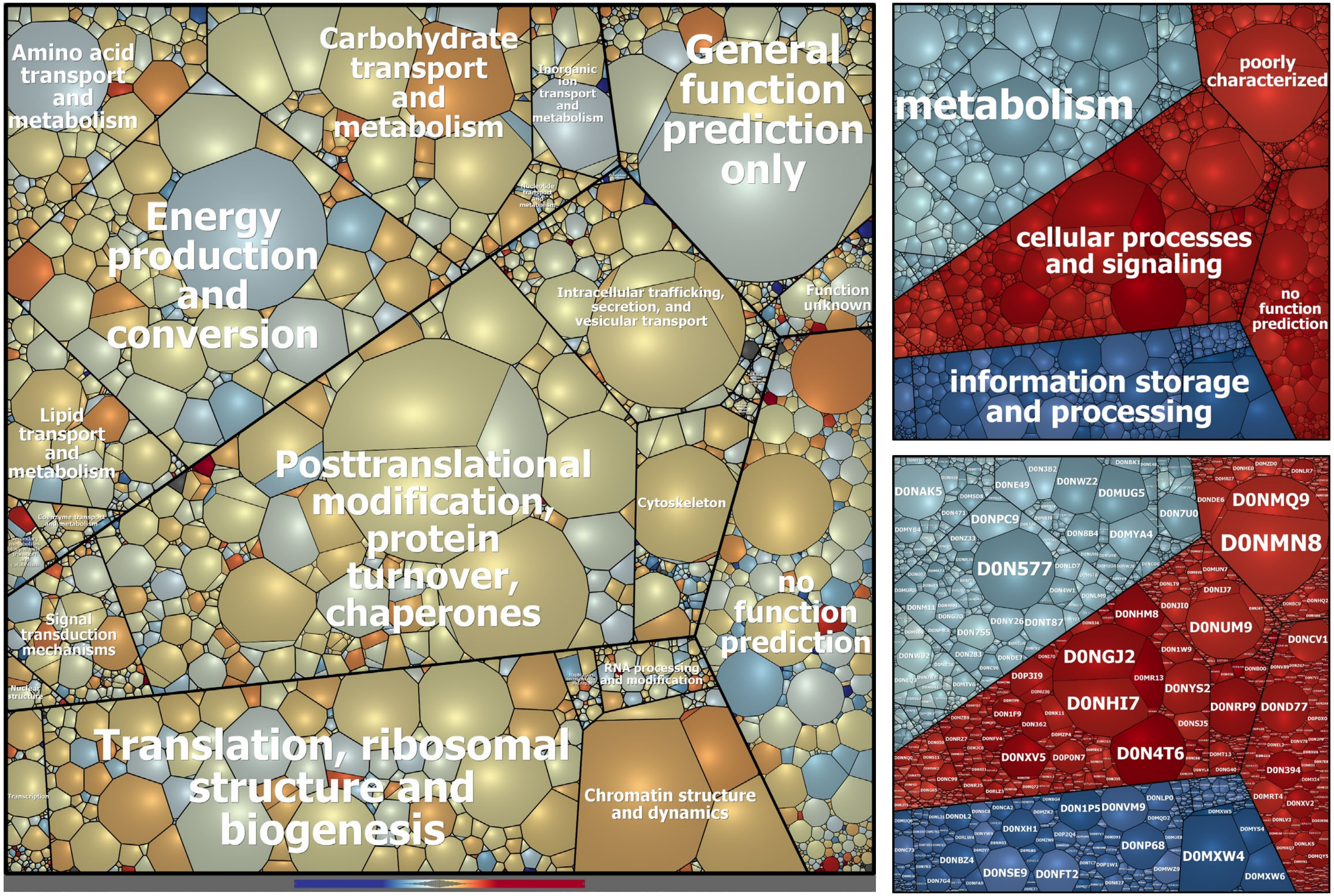

Voronoi treemap visualization of protein expression pattern of *P. infestans* exposed to DMTS.
Relative changes compared to untreated control are color-coded: red – higher amount in treated, blue – higher amount in untreated cultures. Cells sizes were determined by the average protein occurence level within all samples. Proteins are represented by cells, cells are clustered according their functional classification. Classification was done using Prophane 2.0. Grey cells indicate proteins that were not identified.

Effect of **MMTS** exposure on protein pattern of *Phytophthora infestans*

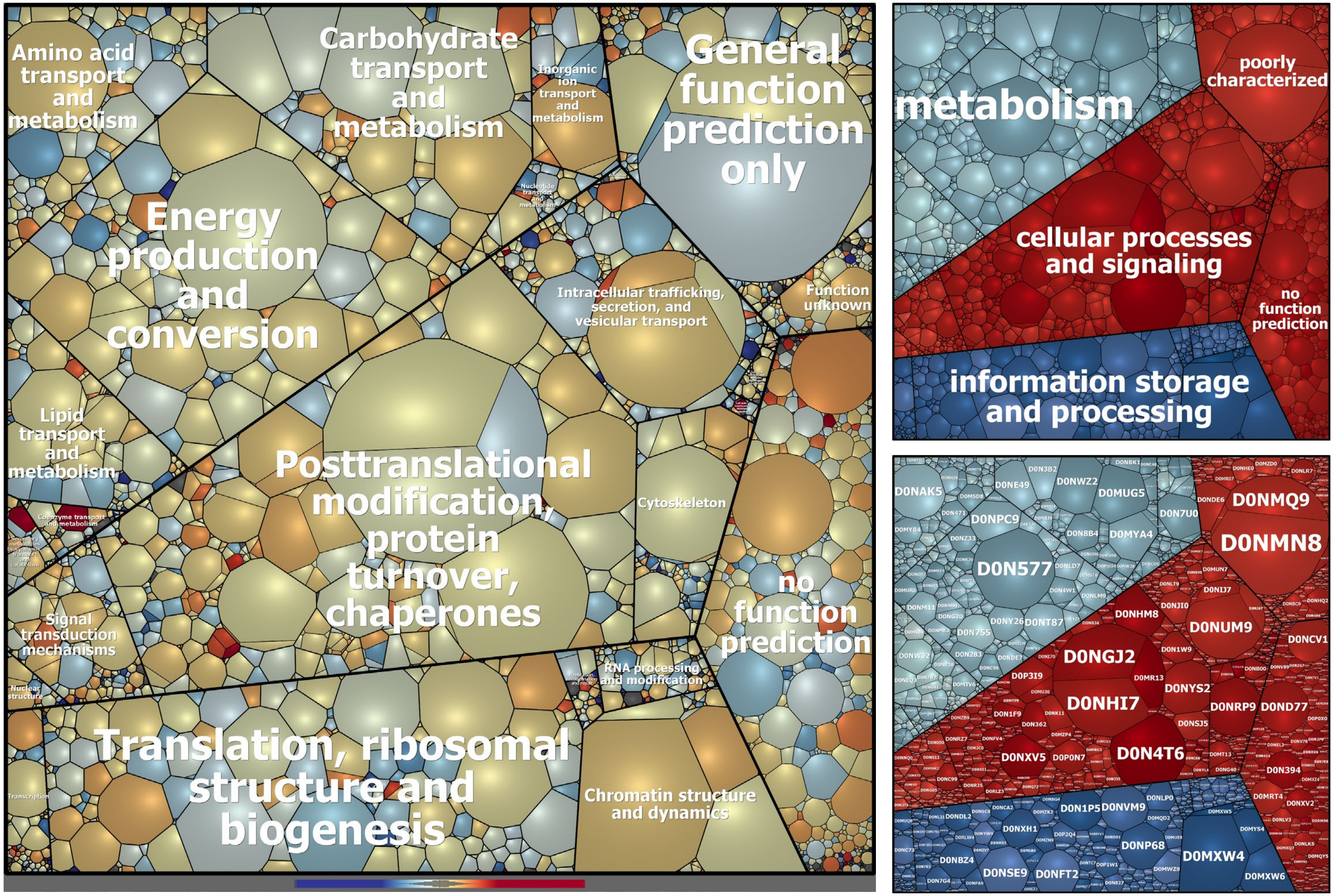

Voronoi treemap visualization of protein expression pattern of *P. infestans* exposed to MMTS.
Relative changes compared to untreated control are color-coded: red – higher amount in treated, blue – higher amount in untreated cultures. Cells sizes were determined by the average protein occurence level within all samples. Proteins are represented by cells, cells are clustered according their functional classification. Classification was done using Prophane 2.0. Grey cells indicate proteins that were not identified.

Effect of **SM** exposure on protein pattern of *Phytophthora infestans*

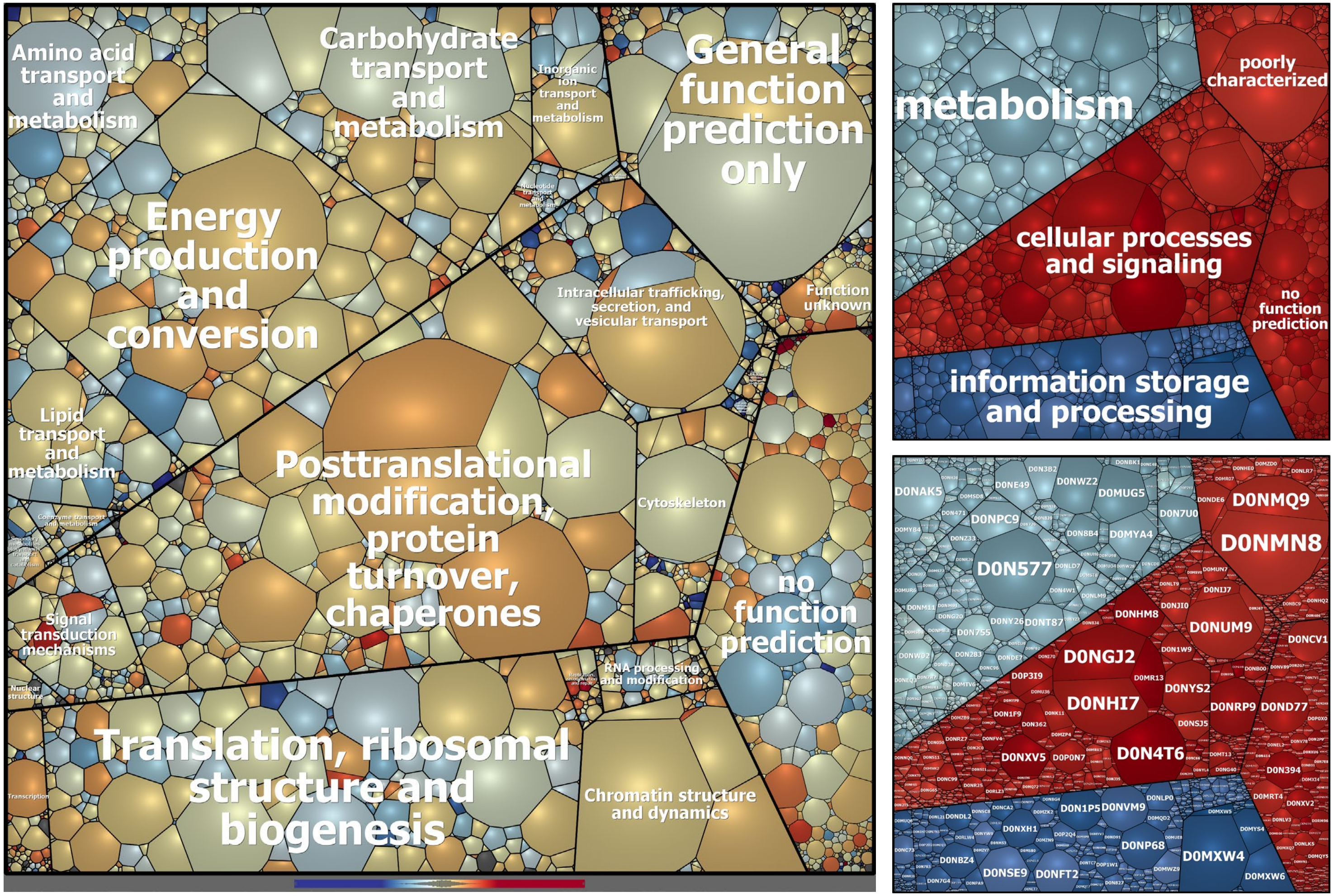

Voronoi treemap visualization of protein expression pattern of *P. infestans* exposed to SM.
Relative changes compared to untreated control are color-coded: red – higher amount in treated, blue – higher amount in untreated cultures. Cells sizes were determined by the average protein occurence level within all samples. Proteins are represented by cells, cells are clustered according their functional classification. Classification was done using Prophane 2.0. Grey cells indicate proteins that were not identified.

Effect of **BMTMS** exposure on protein pattern of *Phytophthora infestans*

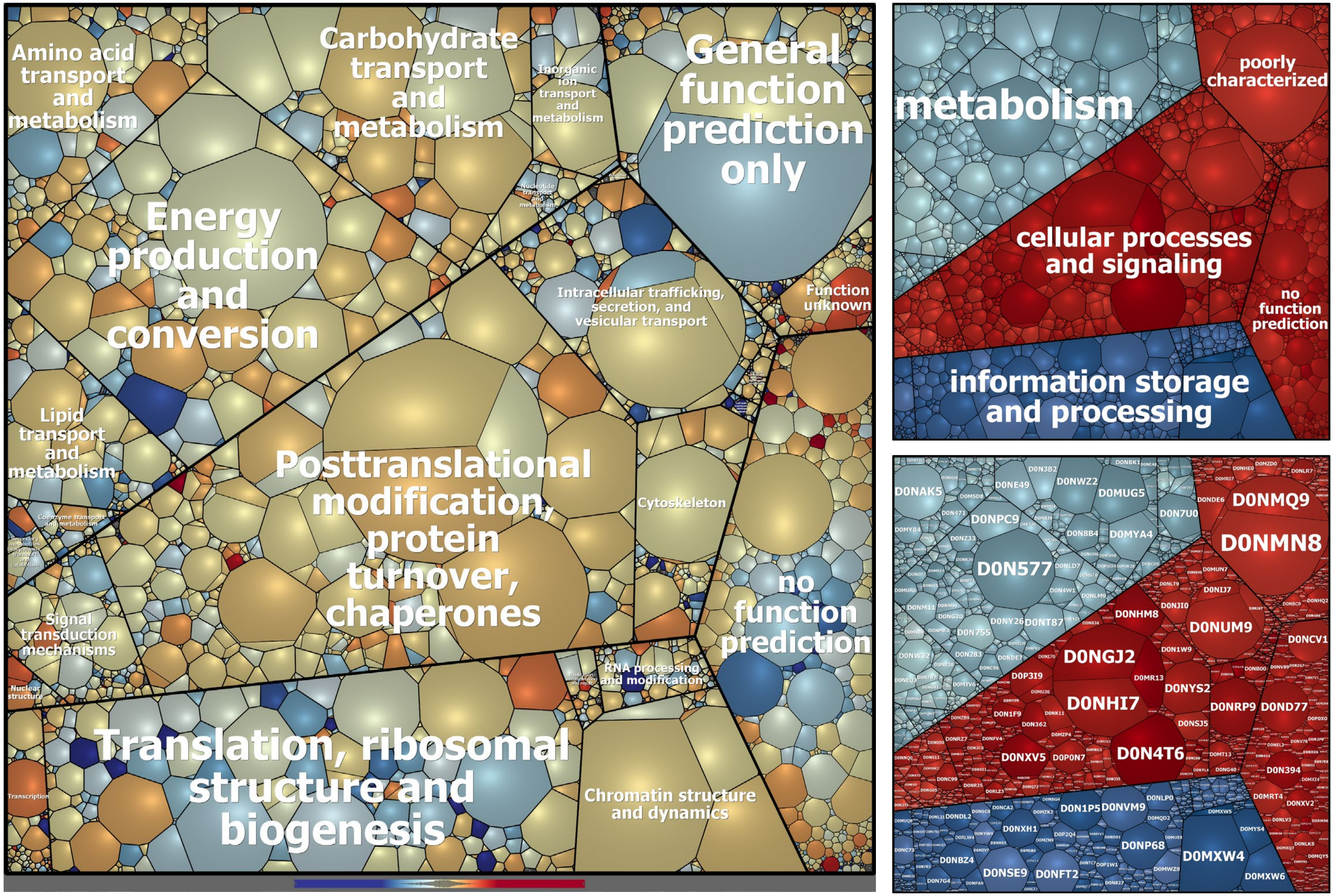

Voronoi treemap visualization of protein expression pattern of *P. infestans* exposed to BMTMS.
Relative changes compared to untreated control are color-coded: red – higher amount in treated, blue – higher amount in untreated cultures. Cells sizes were determined by the average protein occurence level within all samples. Proteins are represented by cells, cells are clustered according their functional classification. Classification was done using Prophane 2.0. Grey cells indicate proteins that were not identified.
